# Comparative Analysis of Coumarin Profiles in Different Parts of *Peucedanum japonicum* and Their Aldo–Keto Reductase Inhibitory Activities

**DOI:** 10.3390/molecules27217391

**Published:** 2022-10-31

**Authors:** Jisu Park, Sunil Babu Paudel, Chang Hyun Jin, Gileung Lee, Hong-Il Choi, Ga-Hee Ryoo, Yun-Seo Kil, Joo-Won Nam, Chan-Hun Jung, Bo-Ram Kim, Min Kyun Na, Ah-Reum Han

**Affiliations:** 1 Advanced Radiation Technology Institute, Korea Atomic Energy Research Institute, Jeollabuk-do, Jeongeup-si 56212, Korea; 2 College of Pharmacy, Chungnam National University, Daejeon 34134, Korea; 3 College of Pharmacy, Yeungnam University, Gyeongsangbuk-do, Gyeongsan-si 38541, Korea; 4 Jeonju AgroBio-Materials Institute, Jeollabuk-do, Jeonju-si 54810, Korea; 5 Natural Product Research Division, Honam National Institute of Biological Resources, Jeollanam-do, Mokpo-si 58762, Korea

**Keywords:** *Peucedanum japonicum*, coumarin, Aldo–keto reductases

## Abstract

*Peucedanum japonicum* (Umbelliferae) is widely distributed throughout Southeast Asian countries. The root of this plant is used in traditional medicine to treat colds and pain, whereas the young leaves are considered an edible vegetable. In this study, the differences in coumarin profiles for different parts of *P. japonicum* including the flowers, roots, leaves, and stems were compared using ultra-performance liquid chromatography time-of-flight mass spectrometry. Twenty-eight compounds were tentatively identified, including three compounds found in the genus *Peucedanum* for the first time. Principal component analysis using the data set of the measured mass values and intensities of the compounds exhibited distinct clustering of the flower, leaf, stem, and root samples. In addition, their anticancer activities were screened using an Aldo–keto reductase (AKR)1C1 assay on A549 human non-small-cell lung cancer cells and the flower extract inhibited AKR1C1 activity. Based on these results, seven compounds were selected as potential markers to distinguish between the flower part versus the root, stem, and leaf parts using an orthogonal partial least-squares discriminant analysis. This study is the first to provide information on the comparison of coumarin profiles from different parts of *P. japonicum* as well as their AKR1C1 inhibitory activities. Taken together, the flowers of *P. japonicum* offer a new use related to the efficacy of overcoming anticancer drug resistance, and may be a promising source for the isolation of active lead compounds.

## 1. Introduction

*Peucedanum japonicum* Thunb. (Umbelliferae) is widely distributed in Korea, Japan, China, and Taiwan [1]. In the records of the traditional medicinal uses of *P. japonicum* in Southeast Asia, the stem and root have been used to treat sore throat [2], whereas the root has been used to treat cough, colds, and headache, and exhibits antipyretic and anti-inflammatory activity [3,4,5]. Coumarins are characteristic compounds of *P. japonicum* and several types of coumarins have been identified in this plant, such as simple coumarins, linear furanocoumarins, linear dihydro furanocoumarins, angular furanocoumarins, angular dihydrofuranocoumarins, linear dihydropyranocoumarins, and angular dihydropyranocoumarins [1,6,7]. Other constituents include chrome, phenolic compounds, and flavonoids [1]. The essential oil extracted from the aerial parts of *P. japonicum* contains α-pinene (24.68%) and β-pinene (66.07%) [5]. Various biological activities reported from the extracts and constituents of *P. japonicum*, such as antioxidant, anti-tyrosinase, anti-microbial, anti-allergic, neuroprotective, antidiabetic, and antiplatelet aggregative effects, have been summarized in Sarkhail et al., 2014 [1]. Other recent reports of in vitro and in vivo activities include anticancer [8], anti-adipogenic [9], antidiabetic [9], anti-osteoporosis [10], anti-allergic lung inflammation [11], antinociceptive [12], prostate contraction inhibitory [13], anti-osteoarthritis [14], antioxidant [15], anti-obesity [16], endothelial dysfunction improvement [17], anti-Alzheimer’s disease [18], corneal damage treatment [19], anti-atopic dermatitis [20], anti-inflammatory [21,22], and anti-influenza virus [23] effects.

Drug resistance is a major obstacle to the overall effectiveness of chemotherapy. Aldo–keto reductases (AKRs), which consist of the AKR1C1-AKR1C4 members, play a pivotal role in nicotinamide-adenine dinucleotide phosphate (NADPH)-dependent reduction and are involved in biosynthesis, intermediate metabolism, and detoxification [24,25]. With respect to the catalytic-dependent function of AKR1C isoforms in cancer chemotherapy, AKRs mediate the reduction of carbonyl groups in anticancer drugs (e.g., daunorubicin, doxorubicin, and oracin) to their inactive metabolites, thus contributing to drug resistance [26,27,28,29]. In addition, AKR1C1 is associated with cisplatin-resistance in colon cancer cell, head and neck squamous cell carcinoma, nasopharyngeal carcinoma, and paediatric T-cell acute lymphoblastic leukemia [30,31,32,33]. It has been recently reported that AKR1C3 mediates chemotherapy resistance in breast cancer and esophageal adenocarcinoma [34,35]. Thus, AKR1C isoforms may be a potential target for preventing the development of anticancer drug resistance. Moreover, AKR1C1 plays a role in regulating the cellular concentration of progesterone by catalyzing the reduction of progesterone to its inactive form, 20-alphahydroxy-progesterone, thus it is also considered a drug target for the prevention of pre-term birth throughout the inhibition of progesterone metabolism mediated by AKR1C1 [36]. 

In our work to identify AKR1C1 inhibitors from natural products to overcome cisplatin-resistance in human non-small lung cancer cells (NSCLC), the chemical composition of the extracts from four parts of *P. japonicum* and their AKR1C1 inhibitory activities in NSCLC (A549) were examined. Ultra-performance liquid chromatography coupled with quadrupole time-of-flight mass spectrometry (UPLC-QTof MS) and multivariate analysis was used to characterize the metabolomic differences between the four extracts.

## 2. Results and Discussion

### 2.1. Identification of Coumarins in the Different Parts of P. japonicum Using UPLC-QTof MS

**Figure 1:**
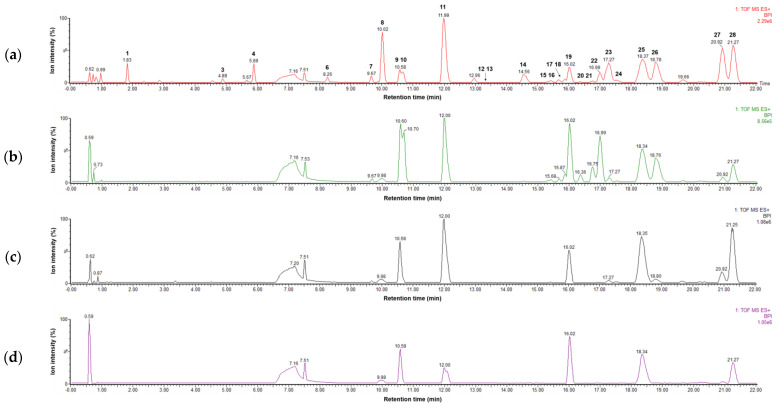
LC–MS base peak ion chromatograms of four extracts representing (**a**) flowers (S3), (**b**) roots (S15), (**c**) leaves (S9), and (**d**) stems (S21) of *Peucedanum japonicum* in positive ion mode (6 eV, ESI^+^).

**Figure 2:**
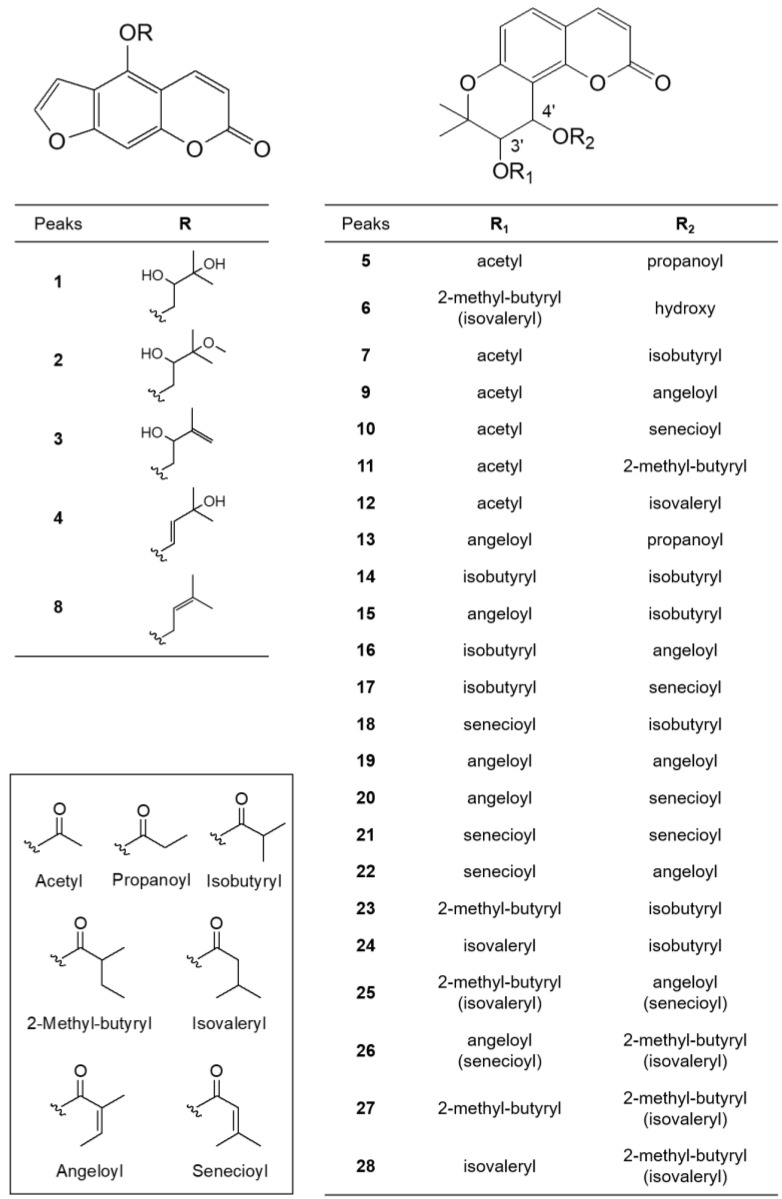
Structures of identified coumarins (Peaks 1–28).

Four parts of *P. japonicum* (flowers, roots, leaves, and stems) were prepared with six samples from each source that were designated as follows: S1–S6 for flower, S13–S18 for root, S7–S12 for leaf, S19–S24 for stem parts. Using UPLC-QTof MS with a gradient solvent system of acetonitrile (containing 0.1% formic acid) and water (containing 0.1% formic acid), the metabolites of the 24 *P. japonicum* samples were separated at high resolution within 22 min in the base peak ion (BPI) chromatogram. The representative BPI chromatograms of the methanol extract of flower, root, leaf, and stem of *P. japonicum* are shown in Figure 1. In these BPI chromatograms, the flower extract exhibited the most peaks and was fractioned using dichloromethane to obtain a chromatogram with increased intensity of coumarins (Figure S1). The mass spectrum of each peak in the BPI chromatogram of the dichloromethane fraction of the *P. japonicum* flowers (Figures S2–S29) was carefully interpreted by analyzing its experimental and theoretical high-resolution MS (the positive mode), error ppm, molecular formula, and MS/MS fragmentation (Table 1), as well as by comparing with existing data for coumarins of the *Peucedanum* species reported in the literature [3,6,37,38,39,40,41,42,43,44,45,46,47,48,49,50,51,52,53]. In addition, UPLC-PDA chromatograms of all the peaks present in the methanol extract of four parts of *P. japonicum* and their UV-Vis spectra between 210–500 nm were shown in Figures S30 and S31. The coumarin structures, which were tentatively identified from the analysis of their MS data, are shown in Figure 2.

Peaks 1–4 and 8 had a common fragment ion for 4-hydroxy-7*H*-furo [3,*2*-*g*][1]benzopyran-7-one (5-hydroxypsoralen) or 9-hydroxy-7*H*-furo [3,*2*-*g*][1]benzopyran-7-one (8-hydroxypsoralen) at *m*/*z* 203.0344 [M − R + H]^+^, which resulted from the loss of different substituents at the C-5 oxygen or the C-8 oxygen [37]. In a review of coumarins isolated from *Peucedanum* species [1], the isolation of linear furanocoumarins, such as bergapten, cnidilin, isoimperatorin, and oxypeucetanin hydrate from *P. japonicum* was reported. Therefore, the linear furanocoumarins in this study were tentatively identified by giving preference to the structure based on the 5-hydroxypsoralen backbone. Peak 1 (*t*_R_ 1.831 min) produced a major molecular ion at *m*/*z* 305.1027 [M + H]^+^ (calculated for C_16_H_17_O_6_^+^, 305.1025) and yielded a fragment ion at *m*/*z* *m*/*z* 203.0344 [M − R + H]^+^, indicating that a 2,3-dihydroxy-3-methylbutyl group was substituted at the 5-OH of 5-hydroxypsoralen. Therefore, peak 1 was tentatively identified as oxypeucedanin hydrate, which has been previously reported in *P. japonicum* [38]. Peak 2 (*t*_R_ 4.712 min) produced a molecular ion at *m*/*z* 319.1223 [M + H]^+^ (calculated for C_17_H_18_O_6_^+^, 319.1223), which was 14 Da less than that of peak 1, indicating the presence of a methoxy group instead of a hydroxyl group. The fragment ions at *m*/*z* 203.0366 [M − R + H]^+^ represent a 2-hydroxy-3-methoxy-3-methylbutyl at the 5-OH of 5-hydroxypsoralen. Thus, it tentatively was identified as oxypeucedanin methanolate, which has been previously reported in *P. ostruthium* [39], although it was first detected in *P. japonicum*. Peaks 3 (*t*_R_ 4.895 min) and 4 (*t*_R_ 5.889 min) produced a molecular ion at *m*/*z* 287.0919 [M + H]^+^ (calculated for C_16_H_15_O_5_^+^, 287.0920), which is 18 Da less than that of peak 1, indicating the loss of a hydroxy group. The fragment ion at *m*/*z* 203.0340 [M − R + H]^+^ indicated the presence of a 2-hydroxy-3methyl-3-butenyl group or a 33-hydroxy-3-methyl-1-butenyl group at the 5-OH of 5-hydroxypsoralen. Therefore, these two peaks were tentatively identified as 5-[(2-hydroxy-3methyl-3-butenyl)oxy]psoralen (pabulenol) and 5-[(3-hydroxy-3-methyl-1-butenyl)oxy]psoralen, respectively, but they are interchangeable. 5-[(2-Hydroxy-3methyl-3-butenyl)oxy]psoralen (pabulenol) has been identified in various species of *Peucedanum* [40], but not in *P. japonicum*, whereas 5-[(3-Hydroxy-3-methyl-1-butenyl)oxy]psoralen has not been reported previously. Peak 8 (*t*_R_ 10.015 min) produced a molecular ion at *m*/*z* 293.0759 [M + Na]^+^ (calculated for C_16_H_14_O_4_Na^+^, 293.0790), which is 16 Da less than that of peak 4, indicating the absence of a hydroxyl group. The fragment ions at *m*/*z* 203.0344 [M − R + H]^+^ represent the presence of a 2-hydroxy-3-methoxy-3-methylbutyl group at the 5-OH of 5-hydroxypsoralen. Thus, it was tentatively identified as isoimperatorin, which has been reported previously in *P. japonicum* [41].

In the ion fragmentation pathways of angular dihydropyranocoumarins, such as khellactone (3,4,5-trihydroxy-2,2-dimethyl-6-chromanacrylic acid δ-lactone), the MS fragment peak of the molecules resulting from a loss of the C-4′ substituent was observed as the main peak and the molecular ion peak due to fragmentation of the C-3′ substituent was small or undetectable [6]. Based on this phenomenon, we concluded that the position of the C-3′ and C-4′ substituents could be determined by MS fragmentation. In this study, molecular ions for angular dihydropyranocoumarins also exhibited characteristic fragment ions of [M-R_2_OH+H]^+^ by the loss of the C-4′ substituent, and [M-R_2_OH-(R_1_-H)+H]^+^ and [M-R_2_OH-(OR_1_-H)+H]^+^ by the loss of the C-3′ substituent. Therefore, in the ion fragmentation pathways of angular dihydropyranocoumarins, fragment ions at *m*/*z* 245.08 [M-R_2_OH-(R_1_-H)+H]^+^ for 5-hydroxy-2,2-dimethyl-3-oxo-6-chromanacrylic acid δ-lactone (jatamasinone) and 227.07 [M-R_2_OH-(OR_1_-H)+H]^+^ for 5-hydroxy-2,2-dimethyl-3,4-didehydro-6-chromanacrylic acid δ-lactone were frequently observed. Peak 5 (*t*_R_ 7.878 min) showed a molecular ion at *m*/*z* 383.1159 [M + Na]^+^ (calculated for C_19_H_20_O_7_Na^+^, 383.1107) and fragment ions at *m*/*z* 287.0963 [M-R_2_OH+H]^+^, 245.0847 [M-R_2_OH-(R_1_-H)+H]^+^ and 227.0740 [M-R_2_OH-(OR_1_-H)+H]^+^, indicating the presence of a propionate group at C-4′ and an acetate group at C-3′. Thus, peak 5 was identified as 3′-*O*-acetyl-4′-*O*-propanoylkhellactone, which was first detected in *P. japonicum*; however, it has also been isolated from *P. prearuptorum* [41]. Peak 6 (*t*_R_ 8.261 min) produced a major molecular ion at *m*/*z* 369.1314 [M + Na]^+^ (calculated for C_19_H_22_O_6_Na^+^, 369.1314) and yielded fragment ions at *m*/*z* 329.1384 [M-R_2_OH+H]^+^ and 245.0814 [M-R_2_OH-(R_1_-H)+H]^+^, indicating the presence of a hydroxyl group at C-4′ and a 2-methyl butanoate or isovalerate group at C-3′. Therefore, peak 6 was tentatively identified as 3′-*O*-(2-methyl-butyryl)-4′-hydroxy khellactone, which was isolated from *P. japonicum* [6] or 3′-*O*-(isovaleryl)-4′-hydroxy khellactone, which was isolated from *P. turgeniifolium* [42], but not *P. japonicum*. Peak 7 (*t*_R_ 9.667 min) showed a molecular ion at *m*/*z* 397.1269 [M + Na]^+^ (calculated for C_20_H_22_O_7_Na^+^, 397.1263), which is 14 Da greater than that of peak 5, with fragment ions at *m*/*z* 287.0924 [M-R_2_OH+H]^+^, showing the presence of the isobutyrate group at C-4′. The fragment ions at *m*/*z* 245.0802 [M-R_2_OH-(R_1_-H)+H]^+^ and 227.0709 [M-R_2_OH-(OR_1_-H)+H]^+^ indicate the presence of the acetate group at C-3′. Thus, peak 7 was identified as 3′-*O*-acetyl-4′-*O*-isobutyryl khellactone, which was first detected in *P. japonicum*; however, it has also been isolated from *P. prearuptorum* [43]. Peaks 9 (*t*_R_ 10.581 min) and 10 (*t*_R_ 10.678 min) yielded identical molecular ions at *m*/*z* 409.1266 [M + Na]^+^ (calculated for C_21_H_22_O_7_Na^+^, 409.1263) and identical fragment ions to one another at *m*/*z* 287.0916 [M-R_2_OH+H]^+^, 245.0814 [M-R_2_OH-(R_1_-H)+H]^+^, and 227.0707 [M-R_2_OH-(OR_1_-H)+H]^+^, indicating the presence of an angeloate or a senecioate group at C-4′ and an acetate group at C-3′. Therefore, peaks 9 and 10 were tentatively identified as 3′-*O*-acetyl-4′-*O*-angeloylkhellactone and 3′-*O*-acetyl-4′-*O*-senecioylkhellactone, respectively, but they are interchangeable. In addition, both compounds were isolated from *P. japonicum* [38]. Peak 11 (*t*_R_ 11.987 min) yielded a major molecular ion at *m*/*z* 411.1419 [M + Na]^+^ (calculated for C_21_H_24_O_7_Na^+^, 411.1420) and a fragment ion at *m*/*z* 287.0921 [M-R_2_OH+H]^+^, suggesting the presence of the 2-methyl butanoate or isovalerate groups at C-4′. The fragment ions at *m*/*z* 245.0820 [M-R_2_OH-(R_1_-H)+H]^+^ and 227.0715 [M-R_2_OH-(OR_1_-H)+H]^+^ indicated that the acetate group was substituted at C-3′. Therefore, peak 11 was tentatively identified as 3′-*O*-acetyl-4′-*O*-(2-methyl butanoate)khellactone [6] or 3′-*O*-acetyl-4′-*O*-isovalerylkhellactone [44], both of which have been isolated from *P. japonicum* [6,44]. Peak 12 (*t*_R_ 13.181 min) produced a major molecular ion at *m*/*z* 423.1532 [M + Na]^+^ (calculated for C_22_H_24_O_7_Na^+^, 423.1420) and yielded fragment ions at *m*/*z* 301.1155 [M-R_2_OH+H]^+^, indicating the presence of the angeloate or a senecioate group at C-4′. The fragment ions at *m*/*z* 245.0878 [M-R_2_OH-(R_1_-H)+H]^+^ and 227.0769 [M-R_2_OH-(OR_1_-H)+H]^+^ suggested a propionate group at C-3′. Thus, peak 12 was tentatively identified as 3′-*O*-propanoyl-4′-*O*-angeloyl khellactone or 3′-*O*-propanoyl-4′-*O*-senecioylkhellactone, both of which have not been previously reported. Peak 13 (*t*_R_ 13.433 min) produced a major molecular ion at *m*/*z* 423.1539 [M + Na]^+^ (calculated for C_22_H_24_O_7_Na^+^, 423.1420) and yielded fragment ions at *m*/*z* 327.1321 [M-R_2_OH+H]^+^, indicating the presence of a propionate group at C-4′. The fragment ions at *m*/*z* 245.0879 [M-R_2_OH-(R_1_-H)+H]^+^ and 227.0769 [M-R_2_OH-(OR_1_-H)+H]^+^ suggested the presence of the angeloate or the senecioate group at C-3′. Thus, peak 13 was tentatively identified as 3′-*O*-angeloyl-4′-*O*-propanoylkhellactone or 3′-*O*-senecioyl-4′-*O*-propanoylkhellactone, both of which have been previously reported in a profiling study of pyranocoumarins in *P. prearuptorum* [45]; however, these molecules were first detected in *P. japonicum.* Peak 14 (*t*_R_ 14.542 min) produced a molecular ion at *m*/*z* 425.1577 [M + Na]^+^, corresponding to the molecular formula C_22_H_26_O_7_Na^+^. The fragment ions at *m*/*z* 315.1229 [M-R_2_OH+H]^+^, 245.0810 [M-R_2_OH-(R_1_-H)+H]^+^, and 227.0710 [M-R_2_OH-(OR_1_-H)+H]^+^ indicated the presence of two isobutyrate groups at C-3′ and C-4′. As a result, peak 14 was tentatively identified as 3′,4′-*O*-diisobutyrylkhellactone. 3′,4′-*O*-Diisobutyrylkhellactone has not been detected in *P. japonicum*; however, it has been isolated from *Glehnia littoralis* [46] and *Phlojodicarpus sibiricus* [47]. Peaks 15 (*t*_R_ 15.319 min) and 18 (*t*_R_ 15.885 min) produced identical molecular ions at *m*/*z* 437.1574 [M + Na]^+^ (calculated for C_23_H_26_O_7_Na^+^, 437.1576) and identical fragmentation patterns. The fragment ion at *m*/*z* 327.1227 [M-R_2_OH+H]^+^ indicated the presence of an isobutyrate group at C-4′. The fragment ions at *m*/*z* 245.0810 [M-R_2_OH-(R_1_-H)+H]^+^ and 227.0710 [M-R_2_OH-(OR_1_-H)+H]^+^ were observed as small peaks; however, they indicated the presence of the angeloate or senecioate group at C-3′. Therefore, peaks 15 and 18 were tentatively identified as 3′-*O*-angeloyl-4′-*O*-isobutyrylkhellactone [45,48] and 3′-*O*-senecioyl-4′-*O*-isobutyrylkhellactone [49], respectively, but they are interchangeable. Both structures have not been found in *P. japonicum*; however, they were reported in *P. prearuptorum* [45,48,49]. Peaks 16 (*t*_R_ 15.442 min) and 17 (*t*_R_ 15.667 min) produced the same molecular ion at *m*/*z* 437.1577 [M + Na]^+^ (calculated for C_23_H_26_O_7_Na^+^, 437.1576) as peaks 15 and 18, but exhibited different a fragment ion at *m*/*z* 315.1229 [M-R_2_OH+H]^+^, suggesting the existence of the angeloate or senecioate group at C-4′. The fragment ions at *m*/*z* 245.0813 [M-R_2_OH-(R_1_-H)+H]^+^ and 227.0702 [M-R_2_OH-(OR_1_-H)+H]^+^ indicated the presence of an isobutyrate group at C-3′. Thus, peaks 16 and 17 were tentatively identified as 3′-*O*- isobutyryl-4′-*O*-angeloylkhellactone and 3′-*O*-isobutyryl-4′-*O*-senecioylkhellactone, respectively, but they are interchangeable. Both structures have not been identified in *P. japonicum*; however, they have been reported in a study profiling pyranocoumarins in *P. prearuptorum* [45]. Peaks 19 (*t*_R_ 16.016 min), 20 (*t*_R_ 16.365 min), 21 (*t*_R_ 16.747 min), and 22 (*t*_R_ 16.993 min) produced identical molecular ions at *m*/*z* 449.1585 [M + Na]^+^ (calculated for C_24_H_26_O_7_Na^+^, 449.1576) and identical fragment ions at *m*/*z* 327.1234 [M-R_2_OH+H]^+^, indicating the presence of the angeloate or senecioate group at C-4′. The fragment ions at *m*/*z* 245.0814 [M-R_2_OH-(R_1_-H)+H]^+^ and *m*/*z* 227.0705 [M-R_2_OH-(OR_1_-H)+H]^+^ resulting from the fragmentation of the C-3′ substituent were observed as small peaks; however, this suggested the presence of the angeloate or senecioate group at C-3′. As a result, peaks 19–22 were tentatively identified as 3′,4′-*O*-diangeloylkhellactone, 3′-*O*-angeloyl-4′-*O*-senecioylkhellactone, 3′,4′-*O*-disenecioylkhellactone, and 3′-*O*-senecioyl-4′-*O*-angeloylkhellactone, respectively, but they are interchangeable. 3′-*O*-senecioyl-4′-*O*-angeloylkhellactone has not been found in *P. japonicum*; however, it has been reported in *P. prearuptorum* [48], whereas 3′,4′-*O*-diangeloylkhellactone [50], 3′-*O*-Angeloyl-4′-*O*-senecioylkhellactone [44], and 3′,4′-*O*-disenecioylkhellactone [51] have been isolated from *P. japonicum*. Peaks 23 (*t*_R_ 17.273 min) and 24 (*t*_R_ 17.542 min) yielded identical molecular ions at *m*/*z* 439.1729 [M + Na]^+^ (calculated for C_23_H_28_O_7_Na^+^, 439.1733). The fragment ions at *m*/*z* 329.1384 [M-R_2_OH+H]^+^ were observed by the loss of an isobutyrate group at C-4′. The fragment ions at *m*/*z* 245.0814 [M-R_2_OH-(R_1_-H)+H]^+^ and 227.0706 [M-R_2_OH-(OR_1_-H)+H]^+^ also indicated the presence of the 2-methyl butyrate or isovalerate group at C-3′. Therefore, peaks 23 and 24 were tentatively identified as 3′-*O*-(2-methyl butyryl)-4′-*O*-isobutyrylkhellactone and 3′-*O*-isovaleryl-4′-*O*-isobutyrylkhellactone, respectively, but they are interchangeable. 3′-*O*-(2-Methyl butyryl)-4′-*O*-isobutyrylkhellactone has been isolated from *P. japonicum* [6] and 3′-*O*-isovaleryl-4′-*O*-isobutyrylkhellactone has not; however, they have been reported in a profiling study of pyranocoumarins in *P. prearuptorum* [45]. Peak 25 (*t*_R_ 18.371 min) showed a molecular ion at *m*/*z* 451.1734 [M + Na]^+^, corresponding to the molecular formula C_24_H_28_O_7_Na^+^. The fragment ions at *m*/*z* 329.1386 [M-R_2_OH+H]^+^ were observed by the loss of the angeloate or senecioate group at C-4′, and the fragment ions at *m*/*z* 245.0815 [M-R_2_OH-(R_1_-H)+H]^+^, and 227.0707 [M-R_2_OH-(OR_1_-H)+H]^+^ indicated the presence of the 2-methyl butyrate or isovalerate group at C-3′. Therefore, peak 25 was tentatively identified as a molecule among the four structures: 3′-*O*-(2-methyl butyryl)-4′-*O*-angeloylkhellactone, 3′-*O*-(2-methyl butyryl)-4′-*O*-senecioyl khellactone, 3′-*O*-isovaleryl-4′-*O*-angeloylkhellactone, and 3′-*O*-isovaleryl-4′-*O*-senecioylkhellactone. 3′-*O*-(2-Methyl butyryl)-4′-*O*-senecioyl khellactone [6], 3′-*O*-isovaleryl-4′-*O*-senecioylkhellactone [51], and 3′-*O*-isovaleryl-4′-*O*-angeloylkhellactone (paeruptorin C) [6] have been isolated from *P. japonicum.* 3′-*O*-(2-Methyl butyryl)-4′-*O*-angeloylkhellactone [52] has not been found in *P. japonicum*; however, it has been identified in *P. prearuptorum* [52]. Peak 26 (*t*_R_ 18.799 min) exhibited the same molecular ion at *m*/*z* 451.1731 [M + Na]^+^ as peak 25, which corresponded to the molecular formula C_24_H_28_O_7_Na^+^; however, the fragment ions at *m*/*z* 327.1237 [M-R_2_OH+H]^+^ indicated that peaks 25 and 26 had substituents different from the substituent at C-4′ from the loss of the 2-methyl butyrate or isovalerate group at C-4′. The fragment ions at *m*/*z* 245.0819 [M-R_2_OH-(R_1_-H)+H]^+^ and 227.0710 [M-R_2_OH-(OR_1_-H)+H]^+^ suggested the presence of an angeloate or senecioate group at C-3′. Therefore, peak 26 was tentatively identified as a molecule among the four structures, 3′-*O*-angeloyl-4′-*O*-(2-methyl butyryl)khellactone, 3′-*O*-angeloyl-4′-*O*-isovaleryl khellactone, 3′-*O*-senecioyl-4′-*O*-(2-methyl butyryl)khellactone, or 3′-*O*-senecioyl-4′-*O*-isovaleryl khellactone. 3′-*O*-Angeloyl-4′-*O*-(2-methyl butyryl)khellactone [53] and 3′-*O*-angeloyl-4′-*O*-isovalerylkhellactone [53] have not been found in *P. japonicum*; however, they have been isolated from *P. prearuptorum* [53]. 3′-*O*-Senecioyl-4′-*O*-(2-methyl butyryl)khellactone [6] and 3′-*O*-senecioyl-4′-*O*-isovalerylkhellactone [3] have been isolated from *P. japonicum.* Peaks 27 (*t*_R_ 20.919 min) and 28 (*t*_R_ 21.268 min) produced the same molecular ion at *m*/*z* 453.1896 [M + Na]^+^ (calculated for C_24_H_30_O_7_Na^+^, 453.1889) and exhibited an identical fragment ion at *m*/*z* 329.1392 [M-R_2_OH+H]^+^, *m*/*z* 245.0817 [M-R_2_OH-(R_1_-H)+H]^+^, and *m*/*z* 227.0708 [M-R_2_OH-(OR_1_-H)+H]^+^, suggesting the presence of a 2-methyl butyrate or isovalerate group at C-4′ and a 2-methyl butyrate or isovalerate group at C-3′. Thus, peaks 27 and 28 were tentatively identified as two molecules among the four structures: 3′,4′-*O*-di(2-methyl butyryl)khellactone, 3′-*O*-(2-methyl butyryl)-4′-*O*- isovalerylkhellactone, 3′-*O*-isovaleryl-4′-*O*-(2-methyl butyryl)khellactone, or 3′,4′-*O*-diisovalerylkhellactone. 3′,4′-*O*-Di(2-methyl butyryl)khellactone [53], 3′-*O*-(2-methyl butyryl)-4′-*O*- isovalerylkhellactone [52], and 3′-*O*-isovaleryl-4′-*O*-(2-methyl butyryl)khellactone [53] have not been found in *P. japonicum*; however, they have been identified previously in *P. prearuptorum* [52,53], whereas 3′,4′-*O*-diisovalerylkhellactone has been isolated from *P. japonicum* [51].

### 2.2. Aldo–Keto Reductases Inhibitory Effects of the Different Parts for P. japonicum

**Figure 3:**
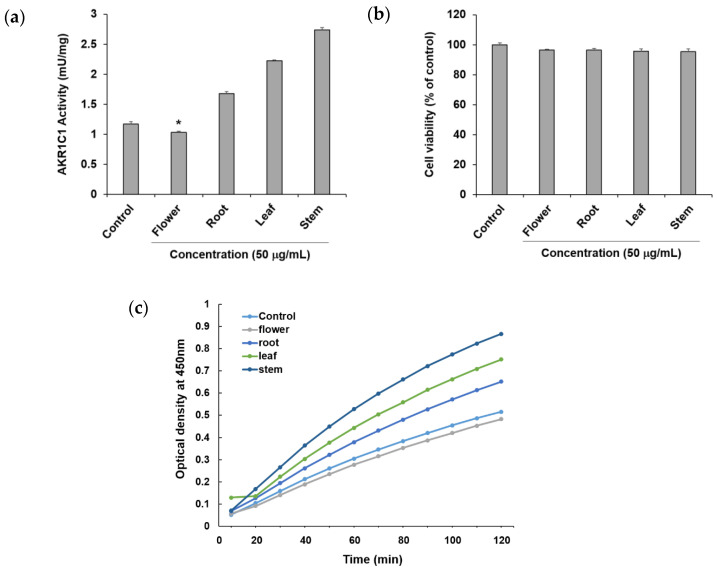
Effects of the methanol extract of four different parts of *Peucedanum japonicum* (50 μg/mL) (**a**) on Aldo–keto reductase (AKR)1C1 activity, (**b**) cell viability, and (**c**) optical density (OD) over time, in A549 human non-small-cell lung cancer cells. Data are presented as means ± SD of three independent experiments. * *p* < 0.05 vs. control.

To confirm the ability of methanol extracts of four different parts of *P. japonicum*, flower (S1–S3), root (S13–S15), leaf (S7–S9), and stem (S19–S21), to circumvent anticancer drug resistance, they were tested using an in vitro AKR1C1 activity assay. Among the four extracts, the flower extract exhibited AKR1C1 activity inhibition of 11.74 ± 0.48% at a concentration of 50 μg/mL without causing cytotoxicity, whereas the other extracts increased AKR1C1 activity (Figure 3a). By observing optical density over time, the flower extract also showed a lower value compared with the control (Figure 3c). Thus, the flower extract was considered a potential natural source for compounds that prevent or overcome anticancer drug resistance in cancer. In the current reports of AKR1C1 inhibitors, non-competitive inhibitors include benzodiazepines, such as medazepam, phthalimide, pyrimidine, and anthranilic acid derivatives [54]. The representative competitive inhibitor of AKR1C1 is 3-bromo-5-phenylsalicylic acid [55]. Efforts for the development of AKR1C1 inhibitors are continuing through molecular docking and in vitro enzymatic studies using a number of synthetic compound derivatives [56,57,58]; however, there have been few reports of AKR1C1 inhibitors developed from natural products [59,60]. Therefore, the flower extract could be a promising source for the isolation of active lead compounds that inhibit AKR1C1 activity.

### 2.3. Multivariate Analysis

**Figure 4:**
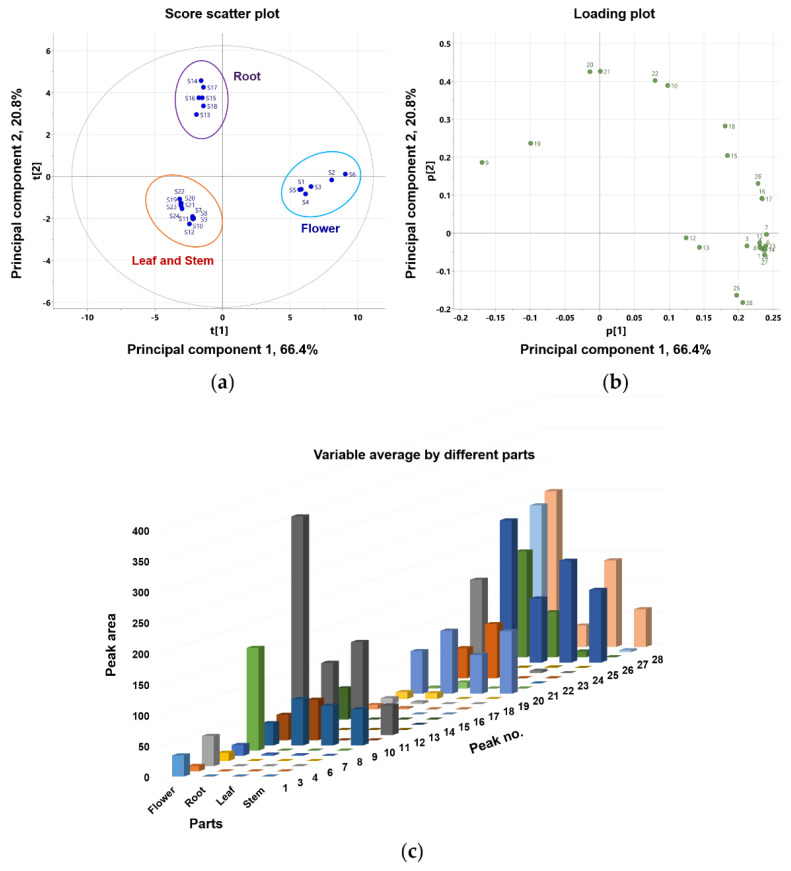
Principal component analysis (PCA) (**a**) score plot and (**b**) loading plot of the metabolome analysis of flower, root, leaf, and stem of *P*. *japonicum*; (**c**) bar chart-showing the variable average for the different parts (flower, root, leaf, and stem) of *P*. *japonicum*.

This study compared the differences in coumarin composition between the flowers, roots, leaves, and stems of *P*. *japonicum*. Using UPLC-QTof MS, the extracts from four different parts were analyzed. Twenty-four samples, six from each part containing 26 coumarins, were included in the multivariate analysis comparison study (peaks 2 and 5 were not included because they were not detected in methanol extracts). Principal component analysis (PCA) clearly distinguished flower (S1–S6), root (S13–S18), leaf (S7–S12), and stem (S19–S24) samples. As shown in Figure 4, leaf (S7–S12) and stem (S19–S24) samples clustered into one large group. PCA was conducted with three principal components (PC1–PC3) that described the variation, 0.913% R2X, and predictive capability, 0.826% Q2. Eigenvalues for PC1 and PC2 were 15.9 and 4.98, respectively, indicating that these first two principal components account for a substantial amount of the data variance. The relatively smaller eigenvalue of PC3 (0.996) led us to select only PC1 and PC2 for further examination. The first two principal components accounted for 87.2% of the variance (66.4% and 20.8% by PC1 and PC2, respectively). The corresponding PCA loading plot (Figure 4b) revealed that most of the peaks were coumarins, except for peaks 9, 10, and 19–21, which were chemical markers responsible for separating the flower sample from the root, leaf, and stem samples. Based on the PCA values, the coumarin profiles for the four parts of *P*. *japonicum* were distinct. Moreover, a variable average by different parts (Figure 4c) also clearly showed that most identified coumarins are abundant in the flower part compared with the root, leaf, and stem parts. Finally, leaves and stems share a chemical profile that is distinct from that of roots and flowers.

**Figure 5:**
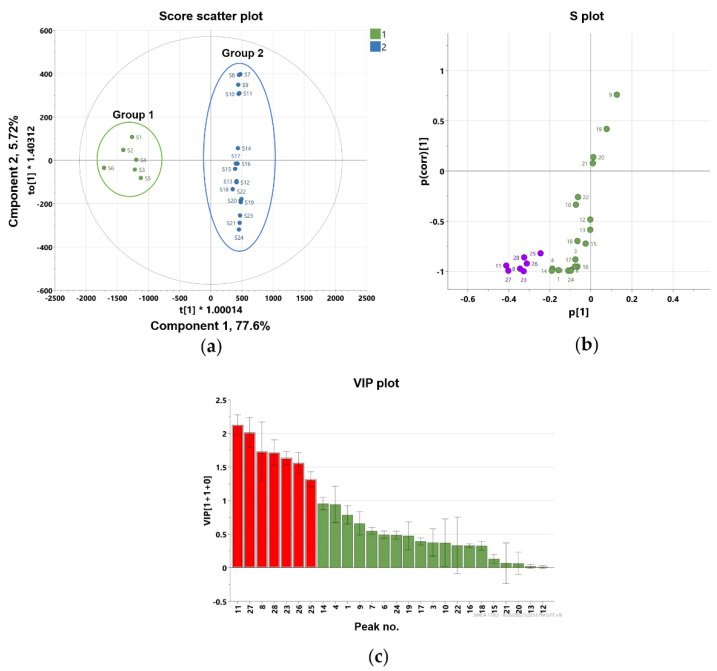
Orthogonal partial least square-discriminant analysis (**a**) score plot and (**b**) S-plot exhibit markers for differentiating flower and root, stem, and leaf parts of *P. japonicum* (purple color shows the most deviated variables from the center), (**c**) the variable importance for the projection plot (VIP-plot) scores of the selected markers (red color shows the variables with VIP values ≥ 1).

To further identify marker compounds responsible for the potency of flower samples, OPLS-DA with a VIP-plot and S-plot were used to compare the groups. Flower samples (S1–S6) were designated as Group 1, while root (S13–S18), leaf (S7–S12), and stem samples (S19–S24) were designated as Group 2. In the OPLS-DA model, flowers versus roots, leaves, and stems were clearly differentiated into two clusters (Figure 5a). The OPLS-DA model had a cumulative R2Y value of 1.00 and a cumulative Q2 value of 0.968. The internal validation of the OPLS-DA model was conducted by a permutation test (n = 200). The intercept values of R2 and Q2 in the permutation test were -0.0066 and -0.399, respectively. All permutations to the left of the R2 and Q2 values were less than the original points to the right (Figure S32). These values indicated that the OPLS-DA model for the analysis was robustly validated and not overfit. Several potential marker compounds capable of distinguishing the two groups were located apart from the center of the corresponding OPLS-DA S-plot (Figure 5b). Seven coumarins, peaks 8, 11, 23, 25–28, were shifted in the same direction as the Group 1 samples on the score scatter plot. The variable importance for the projection plot (VIP-plot, Figure 5c) indicates that these seven coumarins are the primarily responsible markers for discriminating between Group1 and Group 2 with significant VIP values (VIP ≥ 1). This indicates that the Group 1 samples contained higher concentrations of these coumarins compared with the Group 2 samples. Therefore, the composition and relative content of key markers that differentiate between flowers and other parts contribute to distinguishing active and inactive plant parts.

## 3. Materials and Methods

### 3.1. Plant Materials

*P. japonicum* was grown under constant soil conditions in an experimental field located at 35.5114° N latitude by 126.8312° E longitude at the Advanced Radiation Technology Institute, Korea Atomic Energy Research Institute (Jeongeup-si, Jeollabuk-do 56212, Korea) for two years. This plant was bred and identified by Gileung Lee and Hong-Il Choi, co-authors of the study. The different parts of the *P. japonicum* plant (flowers, roots, leaves, and stems) were collected in October 2021, freeze-dried, and stored at –20 °C in polyethylene plastic bags until further use. The voucher specimens were deposited at the Advanced Radiation Technology Institute, Korea Atomic Energy Research Institute.

### 3.2. Sample Preparation

Freeze-dried parts of *P. japonicum* (flowers, roots, leaves, and stems) were ground into powder using a mixer. Six samples were prepared from each part and designated as follows: S1–S6 for flower, S13–S18 for root, S7–S12 for leaf, S19–S24 for stem parts. Then, 1 g of each sample was extracted with 20 mL of methanol in an ultrasonic bath for 1 h and then concentrated. The dried extract (10 mg) was dissolved in 1 mL of methanol and filtered using a 0.20 μm polyvinylidene fluoride filter. The samples were diluted with methanol to a concentration of 500 ppm for further LC–MS analysis. For the evaluation of bioactivity, each dried extract was initially dissolved in dimethyl sulfoxide (DMSO) at a concentration of 50 mg/mL. All extraction and chromatographic solvents used in this study were of analytical grade (J. T. Baker, Phillipsburg, NJ, USA).

### 3.3. UPLC-QTof MS Analysis

A Waters ACQUITY UPLC H-Class system combined with a SYNAPT XS QTof MS (Waters, Milford, MA, USA) was used. Each sample (1 μL) was injected into an ACQUITY UPLC BEH C18 column (100 × 2.1 mm i.d., 1.7 μm) at a flow rate of 0.4 mL/min. The temperature of the column oven was maintained at 40 °C. The mobile phase was composed of 0.1% formic acid in water (*v*/*v*; solvent A) and 0.1% formic acid in acetonitrile (*v*/*v*; solvent B). Gradient elution was carried out as follows: 0–2.0 min, 30% B; 2.0–6.0 min, 30–45% B; 6.0–11.0 min, 45–50% B; 11.0–14.0 min, 50–55% B; 14.0–18.0 min, 55% B; 18.0–21.0 min, 55–60% B; 21.0–25.0 min, 60–100% B; 25.0–27.0 min, 100% B; 27.0–27.1 min, 100–30% B; and 27.1–30.0 min, 30% B. The mass spectrometer was operated in positive ion mode using the following parameters: source temperature, 110 °C; desolvation temperature, 350 °C; capillary voltage, 2.5 kV; cone voltage, 50 V; cone gas flow: 50 L/h; flow rate of desolvation gas (N2), 800 L/h; mass scan range, 100–1200 Da; scan time, 0.5 s. Leucine-enkephalin was used for the lock mass ([M + H]^+^ *m*/*z* 556.2771). Full scan data, MS/MS spectra, accurate mass, and elemental composition were calculated using UNIFI software (version 3.03, Umetrics, Umeå, Sweden).

### 3.4. Aldo–Keto Reductases Activity Assay

The AKR1C1 activity of the extracts was measured using the EZDetect^TM^ Aldo–keto Reductase Activity Assay Kit (Colorimetric) (BioVision Inc., Waltham, MA, USA), based on the manufacturer’s instructions. In this assay, AKR uses a general substrate to convert nicotinamide-adenine dinucleotide phosphate (NADP+) to dihydronicotinamide-adenine dinucleotide phosphate (NADPH). NADPH reacts with the AKR probe and generates a color which is proportional to the activity of AKR in the sample. To determine the effect of the extract on AKR enzyme activity in A549 non-small-cell lung cancer cells (American Type Culture Collection, Rockville, MD, USA), the cells were cultured in a 6-well plate at a density of 5 × 10^5^ cells/mL for 24 h. They were subsequently treated with the extracts at a concentration of 50 μg/mL for an additional 24 h. After the incubation period, the cells were harvested according to the Kit manual for measuring AKR1C1 activity. The assay was performed using a single experiment and the samples were evaluated in triplets.

### 3.5. Statistical and Multivariate Analysis

The data set of the measured mass values and intensities (peak area) for each sample was exported to SIMCA 17.0.2 (Umetrics, Umeå, Sweden) and analyzed by multivariate statistical analysis including PCA and orthogonal partial least-squares discriminant analysis (OPLS-DA). An *S*-plot was used to identify the potential marker compounds that are responsible for the differentiation of the flower part from the root, leaf, and stem parts. Statistical analysis of the biological assay was performed to identify significant differences between the control and test groups (unpaired *t*-test) using GraphPad Prism9 software (GraphPad Prism version 9.0.0 (121) for Mac, San Diego, CA, USA).

## 4. Conclusions

A comparative study was done to identify coumarin composition and AKR1C1 inhibitory activities in different parts of *P. japonicum*. UPLC-QTof MS analysis was used to identify 28 compounds, including three coumarins which have not been reported in the genus *Peucedanum*. Among the methanol extracts of different parts of *P. japonicum*, the flower extract exhibited AKR1C1 activity inhibition in A549 cells at a concentration of 50 μg/mL without cytotoxicity. PCA results showed that the metabolites discriminate among the flower, leaf, stem, and root samples of *P. japonicum* and the OPLS-DA results revealed the responsible markers for discriminating between the active flower extract and the other three inactive parts. These markers were tentatively identified as isoimperatorin (peak 8), 3′-*O*-acetyl-4′-*O*-(2-methyl butanoate)khellactone (or 3′-*O*-acetyl-4′-*O*-isovalerylkhellactone; peak 11), 3′-*O*-(2-methyl butyryl)-4′-*O*-isobutyrylkhellactone (or 3′-*O*-isovaleryl-4′-*O*-isobutyrylkhellactone; peak 23), and compounds having a khellactone structure, in which four substituents, angeloate, senecioate, 2-methyl butyrate, and isovalerate groups, were frequently substituted at C-3 and C-4 (peaks 25–28). Therefore, these markers have the potential to have the ability to inhibit AKR1C1 activity, however further study involving the isolation of these compounds, the determination of their structures and stereochemistry, and the evaluation of their AKR1C1 activity inhibitory effects is required.

## Figures and Tables

**Table 1:** Characterization and tentative identification of coumarins found in *Peucedanum japonicum* using UPLC-QTof MS.

Peak No.	ESI MS *t*_R_ (min)	Observed Mass (*m*/*z*)	Calculated Mass (*m*/*z*)	Error (ppm)	Molecular Formula	Key MS^E^ Fragment Ions (*m*/*z*)	Identification ^1^
1	1.831	305.1027 [M + H]^+^	305.1025	0.2	C_16_H_16_O_6_	631.1793 [2M + Na]^+^ 327.0848 [M + Na]^+^ 203.0344 [M − R + H]^+^	oxypeucedanin hydrate
2	4.712	319.1223 [M + H]^+^	319.1182	4.1	C_17_H_18_O_6_	659.2193 [2M + Na]^+^ 341.1046 [M + Na]^+^ 203.0366 [M − R + H]^+^	oxypeucedanin methanolate
3	4.895	287.0919 [M + H]^+^	287.0920	−0.1	C_16_H_14_O_5_	309.0735 [M + Na]^+^ 203.0340 [M − R + H]^+^	pabulenol
4	5.889	287.0921 [M + H]^+^	287.0920	0.1	C_16_H_14_O_5_	309.0739 [M + Na]^+^ 203.0346 [M − R + H]^+^	5-[(3-hydroxy-3-methyl-1-butenyl)oxy]psoralen
5	7.878	383.1158 [M + Na]^+^	383.1107	5.2	C_19_H_20_O_7_	383.1158 [M + Na]^+^ 287.0963 [M-R_2_OH+H]^+^ 245.0847 [M-R_2_OH-(R_1_-H)+H]^+^ 227.0740 [M-R_2_OH-(OR_1_-H)+H]^+^	3′-*O*-acetyl-4′-*O*-propanoylkhellactone
6	8.261	369.1314 [M + Na]^+^	369.1314	0.0	C_19_H_22_O_6_	715.2726 [2M + Na]^+^ 369.1314 [M + Na]^+^ 329.1384 [M-R_2_OH+H]^+^ 245.0814 [M-R_2_OH-(R_1_-H)+H]^+^	3′- *O*-(2-methyl-butyryl)-4′-hydroxy khellactone (or 3′-*O*-(isovaleryl)-4′-hydroxy khellactone)
7	9.667	397.1269 [M + Na]^+^	397.1263	0.6	C_20_H_22_O_7_	771.2624 [2M + Na]^+^ 397.1269 [M + Na]^+^ 287.0924 [M-R_2_OH+H]^+^ 245.0820 [M-R_2_OH-(R_1_-H)+H]^+^ 227.0709 [M-R_2_OH-(OR_1_-H)+H]^+^	3′-*O*-acetyl-4′-*O*-isobutyryl khellactone
8	10.015	293.0795 [M + Na]^+^	293.0790	0.5	C_16_H_14_O_4_	563.1677 [2M + Na]^+^ 293.0795 [M + Na]^+^ 203.0344 [M – R + H]^+^	isoimpertorin
9	10.581	409.1266 [M + Na]^+^	409.1263	0.3	C_21_H_22_O_7_	795.2621 [2M + Na]^+^ 409.1266 [M + Na]^+^ 287.0916 [M-R_2_OH+H]^+^ 245.0814 [M-R_2_OH-(R_1_-H)+H]^+^ 227.0707 [M-R_2_OH-(OR_1_-H)+H]^+^	3′-*O*-acetyl-4′-*O*-angeloylkhellactone ^a^
10	10.678	409.1270 [M + Na]^+^	409.1263	0.7	C_21_H_22_O_7_	795.2626 [2M + Na]^+^ 409.1270 [M + Na]^+^ 287.0919 [M-R_2_OH+H]^+^ 245.0816 [M-R_2_OH-(R_1_-H)+H]^+^ 227.0708 [M-R_2_OH-(OR_1_-H)+H]^+^	3′-*O*-acetyl-4′-*O*-senecioylkhellactone ^a^
11	11.987	411.1419 [M + Na]^+^	411.1420	−0.1	C_21_H_24_O_7_	799.2952 [2M + Na]^+^ 411.1419 [M + Na]^+^ 287.0921 [M-R_2_OH+H]^+^ 245.0820 [M-R_2_OH-(R_1_-H)+H]^+^ 227.0715 [M-R_2_OH-(OR_1_-H)+H]^+^	3′-*O*-acetyl-4′-*O*-(2-methyl butanoate)khellactone (or 3′-*O*-acetyl-4′-*O*-isovalerylkhellactone)
12	13.181	423.1532 [M + Na]^+^	423.1420	11.2	C_22_H_24_O_7_	823.3156 [2M + Na]^+^ 423.1532 [M + Na]^+^ 301.1155 [M-R_2_OH+H]^+^ 245.0878 [M-R_2_OH-(R_1_-H)+H]^+^ 227.0769 [M-R_2_OH-(OR_1_-H)+H]^+^	3′-*O*-propanoyl-4′-*O*-angeloyl khellactone (or 3′-*O*-propanoyl-4′-*O*-senecioylkhellactone)
13	13.433	423.1539 [M + Na]^+^	423.1420	11.9	C_22_H_24_O_7_	823.3171 [2M + Na]^+^ 423.1539 [M + Na]^+^ 327.1321 [M-R_2_OH+H]^+^ 245.0879 [M-R_2_OH-(R_1_-H)+H]^+^ 227.0769 [M-R_2_OH-(OR_1_-H)+H]^+^	3′-*O*-angeloyl-4′-*O*-propanoylkhellactone (or 3′-*O*-senecioyl-4′-*O*-propanoylkhellactone)
14	14.542	425.1577 [M + Na]^+^	425.1577	0.0	C_22_H_26_O_7_	827.3246 [2M + Na]^+^ 425.1577 [M + Na]^+^ 315.1229 [M-R_2_OH+H]^+^ 245.0813 [M-R_2_OH-(R_1_-H)+H]^+^ 227.0704 [M-R_2_OH-(OR_1_-H)+H]^+^	3′,4′-*O*-diisobutyrylkhellactone
15	15.319	437.1574 [M + Na]^+^	437.1576	−0.2	C_23_H_26_O_7_	851.3203 [2M + Na]^+^ 437.1574 [M + Na]^+^ 327.1227 [M-R_2_OH+H]^+^ 245.0810 [M-R_2_OH-(R_1_-H)+H]^+^ 227.0708 [M-R_2_OH-(OR_1_-H)+H]^+^	3′-*O*-angeloyl-4′-*O*-isobutyrylkhellactone ^b^
16	15.442	437.1577 [M + Na]^+^	437.1576	0.1	C_23_H_26_O_7_	851.3234 [2M + Na]^+^ 437.1577 [M + Na]^+^ 315.1229 [M-R_2_OH+H]^+^ 245.0813 [M-R_2_OH-(R_1_-H)+H]^+^ 227.0702 [M-R_2_OH-(OR_1_-H)+H]^+^	3′-*O*- isobutyryl-4′-*O*-angeloylkhellactone ^c^
17	15.667	437.1581 [M + Na]^+^	437.1576	0.5	C_23_H_26_O_7_	851.3275 [2M + Na]^+^ 437.1581 [M + Na]^+^ 315.1231 [M-R_2_OH+H]^+^ 245.0814 [M-R_2_OH-(R_1_-H)+H]^+^ 227.0704 [M-R_2_OH-(OR_1_-H)+H]^+^	3′-*O*-isobutyryl-4′-*O*-senecioylkhellactone ^c^
18	15.885	437.1584 [M + Na]^+^	437.1576	0.8	C_23_H_26_O_7_	851.3269 [2M + Na]^+^ 437.1584 [M + Na]^+^ 327.1234 [M-R_2_OH+H]^+^ 245.0812 [M-R_2_OH-(R_1_-H)+H]^+^ 227.0704 [M-R_2_OH-(OR_1_-H)+H]^+^	3′-*O*-senecioyl-4′-*O*-isobutyrylkhellactone ^b^
19	16.016	449.1585 [M + Na]^+^	449.1576	0.9	C_24_H_26_O_7_	875.3253 [2M + Na]^+^ 449.1585 [M + Na]^+^ 327.1234 [M-R_2_OH+H]^+^ 245.0814 [M-R_2_OH-(R_1_-H)+H]^+^ 227.0708 [M-OR2-R1-H2O]^+^	3′,4′-*O*-diangeloylkhellactone ^d^
20	16.365	449.1579 [M + Na]^+^	449.1576	0.3	C_24_H_26_O_7_	875.3353 [2M + Na]^+^ 449.1579 [M + Na]^+^ 327.1230 [M-R_2_OH+H]^+^ 245.0810 [M-R_2_OH-(R_1_-H)+H]^+^ 227.0705 [M-OR2-R1-H2O]^+^	3′-*O*-angeloyl-4′-*O*-senecioylkhellactone ^d^
21	16.747	449.1581 [M + Na]^+^	449.1576	0.5	C_24_H_26_O_7_	875.3261 [2M + Na]^+^ 449.1581 [M + Na]^+^ 327.1229 [M-R_2_OH+H]^+^ 245.0809 [M-R_2_OH-(R_1_-H)+H]^+^ 227.0706 [M-OR2-R1-H2O]^+^	3′,4′-*O*-disenecioylkhellactone ^d^
22	16.993	449.1578 [M + Na]^+^	449.1576	0.2	C_24_H_26_O_7_	875.3243 [2M + Na]^+^ 449.1578 [M + Na]^+^ 327.1230 [M-R_2_OH+H]^+^ 245.0811 [M-R_2_OH-(R_1_-H)+H]^+^ 227.0705 [M-OR2-R1-H2O]^+^	3′-*O*-senecioyl-4′-*O*-angeloylkhellactone ^d^
23	17.273	439.1729 [M + Na]^+^	439.1733	−0.4	C_23_H_28_O_7_	855.3560 [2M + Na]^+^ 439.1729 [M + Na]^+^ 329.1384 [M-R_2_OH+H]^+^ 245.0814 [M-R_2_OH-(R_1_-H)+H]^+^ 227.0706 [M-R_2_OH-(OR_1_-H)+H]^+^	3′-*O*-(2-methyl butyryl)-4′-*O*-isobutyrylkhellactone ^e^
24	17.542	439.1734 [M + Na]^+^	439.1733	0.1	C_23_H_28_O_7_	855.3547 [2M + Na]^+^ 439.1734 [M + Na]^+^ 329.1383 [M-R_2_OH+H]^+^ 245.0814 [M-R_2_OH-(R_1_-H)+H]^+^ 227.0706 [M-R_2_OH-(OR_1_-H)+H]^+^	3′-O-isovaleryl-4′-O-isobutyrylkhellactone ^e^
25	18.371	451.1734 [M + Na]^+^	451.1733	0.1	C_24_H_28_O_7_	879.3566 [2M + Na]^+^ 451.1734 [M + Na]^+^ 329.1386 [M-R_2_OH+H]^+^ 245.0815 [M-R_2_OH-(R_1_-H)+H]^+^ 227.0707 [M-R_2_OH-(OR_1_-H)+H]^+^	3′-*O*-(2-methyl butyryl)-4′-*O*-angeloylkhellactone, 3′-*O*-(2-methyl butyryl)-4′-*O*-senecioyl khellactone, 3′-*O*-isovaleryl-4′-*O*-angeloylkhellactone, or 3′-*O*-isovaleryl-4′-*O*-senecioylkhellactone
26	18.799	451.1741 [M + Na]^+^	451.1733	0.8	C_24_H_28_O_7_	879.3578 [2M + Na]^+^ 451.1741 [M + Na]^+^ 327.1237 [M-R_2_OH+H]^+^ 245.0819 [M-R_2_OH-(R_1_-H)+H]^+^ 227.0710 [M-R_2_OH-(OR_1_-H)+H]^+^	3′-*O*-angeloyl-4′-*O*-(2-methyl butyryl)khellactone, 3′-*O*-angeloyl-4′-*O*-isovaleryl khellactone, 3′-*O*-senecioyl-4′-*O*-(2-methyl butyryl)khellactone, or 3′-*O*-senecioyl-4′-*O*-isovaleryl khellactone
27	20.919	453.1896 [M + Na]^+^	453.1889	0.7	C_24_H_30_O_7_	883.3885 [2M + Na]^+^ 453.1896 [M + Na]^+^ 329.1392 [M-R_2_OH+H]^+^ 245.0817 [M-R_2_OH-(R_1_-H)+H]^+^ 227.0708 [M-R_2_OH-(OR_1_-H)+H]^+^	3′,4′-*O*-di-(2-methyl butyryl)khellactone (or 3′-*O*-(2-methyl butyryl)-4′-*O*- isovalerylkhellactone) ^f^
28	21.268	453.1896 [M + Na]^+^	453.1889	0.7	C_24_H_30_O_7_	883.3890 [2M + Na]^+^ 453.1896 [M + Na]^+^ 329.1393 [M-R_2_OH+H]^+^ 245.0818 [M-R_2_OH-(R_1_-H)+H]^+^ 227.0710 [M-R_2_OH-(OR_1_-H)+H]^+^	3′-*O*-isovaleryl-4′-*O*-(2-methyl butyryl)khellactone (or 3′,4′-*O*-diisovalerylkhellactone) ^f^

^1^ Identified molecules with the same superscript letters (a–f) are interchangeable.

## Data Availability

Not applicable.

## Supplementary Materials

The following supporting information can be downloaded at: https://www.mdpi.com/article/10.3390/molecules27217391/s1, Figure S1: LC–MS base peak ion chromatograms of the flowers of *P. japonicum* at positive ion mode (6 eV, ESI+), Figure S2: ESI-QTof-MS spectrum of oxypeucedanin hydrate (peak 1), Figure S3: ESI-QTof-MS spectrum of oxypeucedanin methanolate (peak 2), Figure S4: ESI-QTof-MS spectrum of pabulenol (peak 3), Figure S5: ESI-QTof-MS spectrum of 5-[(3-hydroxy-3-methyl-1-butenyl)oxy]psoralen (peak 4), Figure S6: ESI-QTof-MS spectrum of 3′-O-acetyl-4′-O-propanoylkhellactone (peak 5), Figure S7: ESI-QTof-MS spectrum of 3′- O-(2-methyl-butyryl)-4′-hydroxy khellactone (or 3′-O-(isovaleryl)-4′-hydroxy khellactone) (peak 6), Figure S8: ESI-QTof-MS spectrum of 3′-O-acetyl-4′-O-isobutyryl khellactone (hyuganin D) (peak 7), Figure S9: ESI-QTof-MS spectrum of isoimpertorin (peak 8), Figure S10: ESI-QTof-MS spectrum of 3′-O-acetyl-4′-O-angeloylkhellactone (pteryxin) (peak 9), Figure S11: ESI-QTof-MS spectrum of 3′-O-acetyl-4′-O-senecioylkhellactone (peak 10), Figure S12: ESI-QTof-MS spectrum of 3′-O-acetyl-4′-O-(2-methyl butanoate)khellactone (or 3′-O-acetyl-4′-O-isovalerylkhellactone) (peak 11), Figure S13: ESI-QTof-MS spectrum of 3′-O-propanoyl-4′-O-angeloyl khellactone (or 3′-O-propanoyl-4′-O-senecioylkhellactone) (peak 12), Figure S14: ESI-QTof-MS spectrum of 3′-O-angeloyl-4′-O-propanoylkhellactone (or 3′-O-senecioyl-4′-O-propanoylkhellactone) (peak 13), Figure S15: ESI-QTof-MS spectrum of 3′-O-isobutyryl-4′-O-isobutyrylkhellactone (peak 14), Figure S16: ESI-QTof-MS spectrum of 3′-O-angeloyl-4′-O-isobutyrylkhellactone (peak 15), Figure S17: ESI-QTof-MS spectrum of 3′-O- isobutyryl-4′-O-angeloylkhellactone (peak 16), Figure S18: ESI-QTof-MS spectrum of 3′-O-isobutyryl-4′-O-senecioylkhellactone (peak 17), Figure S19: ESI-QTof-MS spectrum of 3′-O-senecioyl-4′-O-isobutyrylkhellactone (peak 18), Figure S20: ESI-QTof-MS spectrum of 3′-O-angeloyl-4′-O-angeloylkhellactone (paeruptorin B) (peak 19), Figure S21: ESI-QTof-MS spectrum of 3′-O-angeloyl-4′-O-senecioylkhellactone (peak 20), Figure S22: ESI-QTof-MS spectrum of 3′-O-senecioyl-4′-O-senecioylkhellactone (peak 21), Figure S23: ESI-QTof-MS spectrum of 3′-O-senecioyl-4′-O-angeloylkhellactone (peak 22), Figure S24: ESI-QTof-MS spectrum of 3′-O-(2-methyl butyryl)-4′-O-isobutyrylkhellactone (peak23), Figure S25: ESI-QTof-MS spectrum of 3′-O-isovaleryl-4′-O-isobutyrylkhellactone (peak 24), Figure S26: ESI-QTof-MS spectrum of 3′-O-(2-methyl butyryl)-4′-O-angeloylkhellactone [3′-O-(2-methyl butyryl)-4′-O-senecioyl khellactone, 3′-O-isovaleryl-4′-O-angeloylkhellactone (paeruptorin C), or 3′-O-isovaleryl-4′-O-senecioylkhellactone] (peak 25), Figure S27: ESI-QTof-MS spectrum of 3′-O-angeloyl-4′-O-(2-methyl butyryl)khellactone [3′-O-angeloyl-4′-O-isovaleryl khellactone, 3′-O-senecioyl-4′-O-(2-methyl butyryl)khellactone, or 3′-O-senecioyl-4′-O-isovaleryl khellactone] (peak 26), Figure S28: ESI-QTof-MS spectrum of 3′-O-(2-methyl butyryl)-4′-O-(2-methyl butyryl)khellactone (or 3′-O-(2-methyl butyryl)-4′-O- isovalerylkhellactone) (peak 27), Figure S29: ESI-QTof-MS spectrum of 3′-O-isovaleryl-4′-O-(2-methyl butyryl)khellactone (or 3′-O-isovaleryl-4′-O-isovalerylkhellactone) (peak 28), Figure S30: Total scan PDA chromatograms of four extracts representing (**a**) flowers (S3), (**b**) roots (S15), (**c**) leaves (S9), and (**d**) stems (S21) of *Peucedanum japonicum*, Figure S31: UV-Vis spectra of all the peaks present in PDA chromatograms of the methanol extract of four parts of *Peucedanum japonicum* between 200–500 nm, Figure S32: Permutation plot for validation of OPLS-DA obtained from 200 permutation test.

## References

[1] Sarkhail P. (2014). Traditional uses, phytochemistry and pharmacological properties of the genus *Peucedanum*: A review. J. Ethnopharmacol..

[2] Morioka T., Suzui M., Nabandith V., Inamine M., Aniya Y., Nakayama T., Ichiba T., Mori H., Yoshimi N. (2004). The modifying effect of *Peucedanum japonicum*, a herb in the Ryukyu Islands, on azoxymethane-induced colon preneoplastic lesions in male F344 rats. Cancer Lett..

[3] Ikeshiro Y., Mase I., Tomita Y. (1992). Dihydropyranocoumarins from roots of *Peucedanum japonicum*. Phytochemistry.

[4] Lee S.O., Choi S.Z., Lee J.H., Chung S.H., Park S.H., Kang H.C., Yang E.Y., Cho H.J., Lee K. (2004). Antidiabetic coumarin and cyclitol compounds from *Peucedanum japonicum*. Arch. Pharm. Res..

[5] Yang E.J., Kim S.S., Oh T.H., Song G., Kim K.N., Kim J.Y., Lee N.H., Hyun C.G. (2009). *Peucedanum japonicum* and *Citrus unshiu* essential oils inhibit the growth of antibiotic-resistant skin pathogens. Ann. Microbiol..

[6] Hong M.J., Kim J. (2017). Determination of the absolute configuration of khellactone esters from *Peucedanum japonicum* roots. J. Nat. Prod..

[7] Won H.J., Lee S.M., Kim D.-Y., Kwon O.-K., Park M.H., Kim J.-H., Ryu H.W., Oh S.-R. (2019). Rapid securing of reference substances from *Peucedanum japonicum* Thunberg by recycling preparative high-performance liquid chromatography. J. Chromatogr. B.

[8] Kim J.-M., Noh E.-M., Kim H.-R., Kim M.-S., Song H.-K., Lee M., Yang S.-H., Lee G.-S., Moon H.-C., Kwon K.-B. (2016). Suppression of TPA-induced cancer cell invasion by *Peucedanum japonicum* Thunb. extract through the inhibition of PKCα/NF-κB-dependent MMP-9 expression in MCF-7 cells. Int. J. Mol. Med..

[9] Choi R.-Y., Nam S.-J., Ham J.-R., Lee H.-I., Yee S.-T., Kang K.-Y., Seo K.-I., Lee J.-H., Kim M.-J., Lee M.-K. (2016). Anti-adipogenic and anti-diabetic effects of cis-3′,4′-diisovalerylkhellactone isolated from *Peucedanum japonicum* Thunb leaves in vitro. Bioorg. Med. Chem. Lett..

[10] Kim J.-M., Erkhembaatar M., Lee G.S., Lee J.-H., Noh E.-M., Lee M., Song H.-K., Lee C.H., Kwon K.-B., Kim M.S. (2017). *Peucedanum japonicum* Thunb. ethanol extract suppresses RANKL-mediated osteoclastogenesis. Exp. Ther. Med..

[11] Chun J.M., Lee A.R., Kim H.S., Lee A.Y., Gu G.J., Moon B.C., Kwon B.-I. (2018). *Peucedanum japonicum* extract attenuates allergic airway inflammation by inhibiting Th2 cell activation and production of pro-inflammatory mediators. J. Ethnopharmacol..

[12] Kim S.H., Jong H.S., Yoon M.H., Oh S.H., Jung K.T. (2017). Antinociceptive effect of intrathecal sec-*O*-glucosylhamaudol on the formalin-induced pain in rats. Korean J. Pain.

[13] Suzuki T., Otsuka A., Ito Y., Yamada S., Miyake H., Ozono S. (2018). Isosamidin, an extract of *Peucedanum japonicum*, inhibits phenylephrine-mediated contractions of the human prostate in vitro. Phytother. Res..

[14] Chun J.M., Lee A.Y., Kim J.S., Choi G., Kim S.H. (2018). Protective Effects of *Peucedanum japonicum* extract against osteoarthritis in an animal model using a combined systems approach for compound-target prediction. Nutrients.

[15] Taira J., Ogi T. (2019). Induction of antioxidant protein HO-1 through Nrf2-ARE signaling due to pteryxin in *Peucedanum japonicum* Thunb in RAW264.7 macrophage cells. Antioxidants.

[16] Hossin A.Y., Inafuku M., Oku H. (2019). Dihydropyranocoumarins exerted anti-obesity activity in vivo and its activity was enhanced by nanoparticulation with polylactic-co-glycolic acid. Nutrients.

[17] Do M.H., Lee J.H., Ahn J., Hong M.J., Kim J., Kim S.Y. (2020). Isosamidin from *Peucedanum japonicum* roots prevents methylglyoxal-induced glucotoxicity in human umbilical vein endothelial cells via suppression of ROS-mediated Bax/Bcl-2. Antioxidants.

[18] Heo J.H., Eom B.H., Ryu H.W., Kang M.-G., Park J.E., Kim D.-Y., Kim J.-H., Park D., Oh S.-R., Kim H. (2020). Acetylcholinesterase and butyrylcholinesterase inhibitory activities of khellactone coumarin derivatives isolated from *Peucedanum japonicum* Thurnberg. Sci. Rep..

[19] Kang W.S., Choi H., Lee K.H., Kim E., Kim K.J., Na C.S., Kim S. (2021). *Peucedanum japonicum* Thunberg and its active components mitigate oxidative stress, inflammation and apoptosis after urban particulate matter-induced ocular surface damage. Antioxidants.

[20] Gil T.Y., Jin B.R., An H.J. (2022). *Peucedanum japonicum* Thunberg alleviates atopic dermatitis-like inflammation via STAT/MAPK signaling pathways in vivo and in vitro. Mol. Immunol..

[21] Hwang D., Ryu H.W., Park J.-W., Kim J.-H., Kim D.-Y., Oh J.-H., Kwon O.-K., Han S.-B., Ahn K.-S. (2022). Effects of 3′-isovaleryl-4′-senecioylkhellactone from *Peucedanum japonicum* Thunberg on PMA-stimulated inflammatory response in A549 human lung epithelial cells. J. Microbiol. Biotechnol..

[22] Gil T.Y., Jin B.R., Lee J.H., An H.J. (2022). In vitro and in vivo experimental investigation of anti-inflammatory effects of *Peucedanum japonicum* aqueous extract by suppressing the LPS-induced NF-κB/MAPK JNK pathways. Am. J. Chin. Med..

[23] Kanazawa R., Morimoto R., Horio Y., Sumitani H., Isegawa Y. (2022). Inhibition of influenza virus replication by Apiaceae plants, with special reference to *Peucedanum japonicum* (Sacna) constituents. J. Ethnopharmacol..

[24] Zeng C.M., Chang L.L., Ying M.D., Cao J., He Q.J., Zhu H., Yang B. (2017). Aldo-keto reductase AKR1C1-AKR1C4: Functions, regulation, and intervention for anti-cancer therapy. Front. Pharmacol..

[25] Penning T.M., Jonnalagadda S., Trippier P.C., Rižner T.L. (2021). Aldo-keto reductases and cancer drug resistance. Pharmacol. Rev..

[26] Barski O.A., Tipparaju S.M., Bhatnagar A. (2008). The aldo-keto reductase superfamily and its role in drug metabolism and detoxification. Drug. Metab. Rev..

[27] Minotti G., Menna P., Salvatorelli E., Cairo G., Gianni L. (2004). Anthracyclines: Molecular advances and pharmacologie developments in antitumor activity and cardiotoxicity. Pharmacol. Rev..

[28] Hofman J., Malcekova B., Skarka A., Novotna E., Wsol V. (2014). Anthracycline resistance mediated by reductive metabolism in cancer cells: The role of aldo-keto reductase 1C3. Toxicol. Appl. Pharmacol..

[29] Wsol V., Szotakova B., Martin H.J., Maser E. (2007). Aldo-keto reductases (AKR) from the AKR1C subfamily catalyze the carbonyl reduction of the novel anticancer drug oracin in man. Toxicology.

[30] Matsunaga T., Hojo A., Yamane Y., Endo S., El-Kabbani O., Hara A. (2013). Pathophysiological roles of aldo-keto reductases (AKR1C1 and AKR1C3) in development of cisplatin resistance in human colon cancers. Chem. Biol. Interact..

[31] Chang W.M., Chang Y.C., Yang Y.C., Lin S.K., Chang P.M., Hsiao M. (2019). AKR1C1 controls cisplatin-resistance in head and neck squamous cell carcinoma through cross-talk with the STAT1/3 signaling pathway. J. Exp. Clin. Cancer Res..

[32] Zhou C., Shen G., Yang F., Duan J., Wu Z., Yang M., Liu Y., Du X., Zhang X., Xiao S. (2020). Loss of AKR1C1 is a good prognostic factor in advanced NPC cases and increases chemosensitivity to cisplatin in NPC cells. J. Cell Mol. Med..

[33] Bortolozzi R., Bresolin S., Rampazzo E., Paganin M., Maule F., Mariotto E., Boso D., Minuzzo S., Agnusdei V., Viola G. (2018). AKR1C enzymes sustain therapy resistance in paediatric T-ALL. Br. J. Cancer..

[34] Kobayashi M., Yonezawa A., Takasawa H., Nagao Y., Iguchi K., Endo S., Ikari A., Matsunaga T. (2022). Development of cisplatin resistance in breast cancer MCF7 cells by up-regulating aldo-keto reductase 1C3 expression, glutathione synthesis and proteasomal proteolysis. J. Biochem..

[35] Zhou C., Wang Z., Li J., Wu X., Fan N., Li D., Liu F., Plum P.S., Hoppe S., Hillmer A.M. (2021). Aldo-keto reductase 1C3 mediates chemotherapy resistance in esophageal adenocarcinoma via ROS detoxification. Cancers.

[36] Ji Q., Aoyama C., Nien Y.D., Liu P.I., Chen P.K., Chang L., Stanczyk F.Z., Stolz A. (2004). Selective loss of AKR1C1 and AKR1C2 in breast cancer and their potential effect on progesterone signaling. Cancer Res..

[37] Shi H., Chang Y.Q., Feng X., Yang G.Y., Zheng Y.G., Zheng Q., Zhang L.L., Zhang D., Guo L. (2022). Chemical comparison and discrimination of two plant sources of *Angelicae dahuricae* Radix, *Angelica dahurica* and *Angelica dahurica* var. *formosana*, by HPLC-Q/TOF-MS and quantitative analysis of multiple components by a single marker. Phytochem. Anal..

[38] Chen I.S., Chang C.T., Sheen W.S., Teng C.M., Tsai I.L., Duh C.Y., Ko F.N. (1996). Coumarins and antiplatelet aggregation constituents from Formosan *Peucedanum japonicum*. Phytochemistry.

[39] Reisch J., Khaled S.A., Szendrei K., Novak I. (1975). Natural product chemistry. 51. 5-Alkoxy-furanocoumarins from *Peucedanum ostruthium*. Phytochemistry.

[40] Varga E., Simokovics J., Szendrei K., Reisch J. (1979). Furanocoumarins and chromones from the fruits of *Peucedanum ostrutbium* L. (Koch) (Umbelliferae). Fitoterapia.

[41] Lee J., Lee Y.J., Kim J., Bang O.-S. (2015). Pyranocoumarins from root extracts of *Peucedanum praeruptorum* dunn with multidrug resistance reversal and anti-inflammatory activities. Molecules.

[42] Sun H., Lin Z., Niu F., Ding J. (1981). Studies on Chinese herbs in Umbelliferae. II. New coumarins in *Peucedanum turgeniifolium* Wolff. Yunnan Zhiwu Yanjiu.

[43] Song Y.-L., Jing W.-H., Tu P.-F., Wang Y.-T. (2014). Enantiomeric separation of angular-type pyranocoumarins from Peucedani Radix using AD-RH chiral column. Nat. Prod. Res..

[44] Taira N., Nugara R.N., Inafuku M., Takara K., Ogi T., Ichiba T., Iwasaki H., Okabe T., Oku H. (2017). In vivo and in vitro anti-obesity activities of dihydropyranocoumarins derivatives from *Peucedanum japonicum* Thunb. J. Funct. Foods..

[45] Yang W., Li Y., Kang C., Zhao H., Xiang L., Li C., Wang Q. (2017). Sodiation-based in-source collision for profiling of pyranocoumarins in Radix Peucedani (Qianhu): Utility of sodium adducts’ stability with in-source collision. J. Mass Spectrom..

[46] Lee J.W., Lee C., Jin Q., Yeon E.T., Lee D., Kim S.-Y., Han S.B., Hong J.T., Lee M.K., Hwang B.Y. (2014). Pyranocoumarins from *Glehnia littoralis* inhibit the LPS-induced NO production in macrophage RAW 264.7 cells. Bioorg. Med. Chem. Lett..

[47] Olennikov D.N., Fedorov I.A., Kashchenko N.I., Chirikova N.K., Vennos C. (2019). Khellactone derivatives and other phenolics of *Phlojodicarpus sibiricus* (Apiaceae): HPLC-DAD-ESI-QQQ-MS/MS and HPLC-UV profile, and antiobesity potential of dihydrosamidin. Molecules.

[48] Wang X.-Y., Li J.-F., Jian Y.-M., Wu Z., Fang M.-J., Qiu Y.-K. (2015). On-line comprehensive two-dimensional normal-phase liquid chromatography × reversed-phase liquid chromatography for preparative isolation of *Peucedanum praeruptorum*. J. Chromatogr. A.

[49] Li X.-M., Jiang X.-J., Yang K., Wang L.-X., Wen S.-Z., Wang F. (2016). Prenylated Coumarins from *Heracleum stenopterum*, *Peucedanum praeruptorum*, *Clausena lansium*, and *Murraya paniculata*. Nat. Prod. Bioprospect..

[50] Hata K., Kozawa M., Ikeshiro Y., Yen K.-Y. (1968). New coumarins isolated from the root of *Peucedanum formosanum* and *Peucedanum japonicum*. Yakugaku Zasshi.

[51] Jong T.T., Hwang H.C., Jean M.Y., Wu T.S., Teng C.M. (1992). An antiplatelet aggregation principle and X-ray structural analysis of cis-khellactone diester from *Peucedanum japonicum*. J. Nat. Prod..

[52] Song Y., Song Q., Liu Y., Li J., Wan J.-B., Wang Y., Jiang Y., Tu P. (2017). Integrated work-flow for quantitative metabolome profiling of plants, Peucedani Radix as a case. Anal. Chim. Acta.

[53] Lv H., Luo J., Wang X., Kong L. (2013). Application of UPLC-Quadrupole-TOF-MS coupled with recycling preparative HPLC in isolation and preparation of coumarin isomers with similar polarity from *Peucedanum praeruptorum*. Chromatographia.

[54] Brozic P., Cesar J., Kovac A., Davies M., Johnson A.P., Fishwick C.W.G., Lanisnik Rizner T., Gobec S. (2009). Derivatives of pyrimidine, phthalimide and anthranilic acid as inhibitors of human hydroxysteroid dehydrogenase AKR1C1. Chem. Biol. Interact.

[55] El-Kabbani O., Scammells P.J., Gosling J., Dhagat U., Endo S., Matsunaga T., Soda M., Hara A. (2009). Structure-guided design, synthesis, and evaluation of salicylic acid-based inhibitors targeting a selectivity pocket in the active site of human 20alpha-hydroxysteroid dehydrogenase (AKR1C1). J. Med. Chem..

[56] Verma P., Hassan M.I., Singh A., Singh I.K. (2021). Design and development of novel inhibitors of aldo-ketoreductase 1C1 as potential lead molecules in treatment of breast cancer. Mol. Cell Biochem..

[57] He S., Liu Y., Chu X., Li Q., Lyu W., Liu Y., Xing S., Feng F., Liu W., Guo Q. (2022). Discovery of novel aldo-keto reductase 1C3 inhibitors as chemotherapeutic potentiators for cancer drug resistance. ACS Med. Chem. Lett..

[58] Kljun J., Pavlič R., Hafner E., Lipec T., Moreno-Da Silva S., Tič P., Turel I., Büdefeld T., Stojan J., Rižner T.L. (2022). Ruthenium complexes show potent inhibition of AKR1C1, AKR1C2, and AKR1C3 enzymes and anti-proliferative action against chemoresistant ovarian cancer cell line. Front Pharmacol..

[59] Zeng C., Zhu D., You J., Dong X., Yang B., Zhu H., He Q. (2019). Liquiritin, as a natural inhibitor of AKR1C1, could interfere with the progesterone metabolism. Front. Physiol..

[60] Fu Z., Li S., Liu J., Zhang C., Jian C., Wang L., Zhang Y., Shi C. (2022). Natural product alantolactone targeting AKR1C1 suppresses cell proliferation and metastasis in non-small-cell lung cancer. Front. Pharmacol..

