# Supplementary material for: Comparative Analysis of Coumarin Profiles in Different Parts of Peucedanum japonicum and Their Aldo–Keto Reductase Inhibitory Activities
Source: Molecules. 2022 Oct 31;27(21):7391. doi: 10.3390/molecules27217391 (PMC9657185; doi:10.3390/molecules27217391)
Supplement: Supplementary file 1 [file molecules-27-07391-s001.zip › molecules-1965465-supplementary.pdf]

Supporting Information

Comparative analysis of coumarin profiles in different parts of *Peucedanum japonicum* and their aldo-keto reductase inhibitory activities

Jisu Park [1,2], Sunil Babu Paudel [3], Chang Hyun Jin [1], Gileung Lee [1], Hong-Il Choi [1], Ga-Hee Ryoo [1], Yun-Seo Kil [3], Joo-Won Nam [3], Chan-Hun Jung [4], Bo-Ram Kim [5], MinKyun Na [2], Ah-Reum Han [1,*]

1 Advanced Radiation Technology Institute, Korea Atomic Energy Research Institute, Jeongeup-si, Jeollabuk-do 56212, Republic of Korea; parkjs94@kaeri.re.kr (J.P.); chjin@kaeri.re.kr (C.H.J.); glee@kaeri.re.kr (G.L.); hichoi@kaeri.re.kr (H.-I.C.); ghryoo@kaeri.re.kr (G.H.R.)
2 College of Pharmacy, Chungnam National University, Daejeon 34134, Republic of Korea; mkna@cnu.ac.kr (M.N)
3 College of Pharmacy, Yeungnam University, Gyeongsan-si, Gyeongsangbukdo 38541, Republic of Ko-rea; phrsunil@gmail.com (S.B.P.); yskil@yu.ac.kr (Y.-S.K.); jwnam@yu.ac.kr (J.-W.N.)
4 Jeonju AgroBio-Materials Institute, Jeonju-si, Jeollabuk-do 54810, Republic of Korea; chjung@jami.re.kr (C.-H.J.)
5 Natural Product Research Division, Honam National Institute of Biological Resources, Mokpo-si, Jeollanam-do 58762, Republic of Korea; boram0307@hnibr.re.kr (B.-R.K.)
* Correspondence: arhan@kaeri.re.kr (A.-R.H.), Tel.: +82-63-570-3167 (A.-R.H.)

CONTENTS

Figure S1. LC–MS base peak ion chromatograms of the flowers of *P. japonicum* at positive ion mode (6 eV, ESI+)
Figure S2. ESI-QTof-MS spectrum of oxypeucedanin hydrate (peak 1)
Figure S3. ESI-QTof-MS spectrum of oxypeucedanin methanolate (peak 2)
Figure S4. ESI-QTof-MS spectrum of pabulenol (peak 3)
Figure S5. ESI-QTof-MS spectrum of 5-[(3-hydroxy-3-methyl-1-butenyl)oxy]psoralen (peak 4)
Figure S6. ESI-QTof-MS spectrum of 3'-O-acetyl-4'-O-propanoylkhellactone (peak 5)
Figure S7. ESI-QTof-MS spectrum of 3'- O-(2-methyl-butyryl)-4'-hydroxy khellactone (or 3'-O-(isovaleryl)-4'-hydroxy khellactone) (peak 6)
Figure S8. ESI-QTof-MS spectrum of 3'-O-acetyl-4'-O-isobutyryl khellactone (hyuganin D) (peak 7)
Figure S9. ESI-QTof-MS spectrum of isoimpertorin (peak 8)
Figure S10. ESI-QTof-MS spectrum of 3'-O-acetyl-4'-O-angeloylkhellactone (pteryxin) (peak 9)
Figure S11. ESI-QTof-MS spectrum of 3'-O-acetyl-4'-O-senecioylkhellactone (peak 10)
Figure S12. ESI-QTof-MS spectrum of 3'-O-acetyl-4'-O-(2-methyl butanoate)khellactone (or 3'-O-acetyl-4'-O-isovalerylkhellactone) (peak 11)
Figure S13. ESI-QTof-MS spectrum of 3'-O-propanoyl-4'-O-angeloyl khellactone (or 3'-O-propanoyl-4'-O-senecioylkhellactone) (peak 12)
Figure S14. ESI-QTof-MS spectrum of 3'-O-angeloyl-4'-O-propanoylkhellactone (or 3'-O-senecioyl-4'-O-propanoylkhellactone) (peak 13)
Figure S15. ESI-QTof-MS spectrum of 3'-O-isobutyryl-4'-O-isobutyrylkhellactone (peak 14)
Figure S16. ESI-QTof-MS spectrum of 3'-O-angeloyl-4'-O-isobutyrylkhellactone (peak 15)
Figure S17. ESI-QTof-MS spectrum of 3'-O- isobutyryl-4'-O-angeloylkhellactone (peak 16)
Figure S18. ESI-QTof-MS spectrum of 3'-O-isobutyryl-4'-O-senecioylkhellactone (peak 17)
Figure S19. ESI-QTof-MS spectrum of 3'-O-senecioyl-4'-O-isobutyrylkhellactone (peak 18)
Figure S20. ESI-QTof-MS spectrum of 3'-O-angeloyl-4'-O-angeloylkhellactone (paeruptorin B) (peak 19)
Figure S21. ESI-QTof-MS spectrum of 3'-O-angeloyl-4'-O-senecioylkhellactone (peak 20)
Figure S22. ESI-QTof-MS spectrum of 3'-O-senecioyl-4'-O-senecioylkhellactone (peak 21)
Figure S23. ESI-QTof-MS spectrum of 3'-O-senecioyl-4'-O-angeloylkhellactone (peak 22)
Figure S24. ESI-QTof-MS spectrum of 3'-O-(2-methyl butyryl)-4'-O-isobutyrylkhellactone (peak23)
Figure S25. ESI-QTof-MS spectrum of 3'-O-isovaleryl-4'-O-isobutyrylkhellactone (peak 24)
Figure S26. ESI-QTof-MS spectrum of 3'-O-(2-methyl butyryl)-4'-O-angeloylkhellactone [3'-O-(2-methyl butyryl)-4'-O-senecioyl khellactone, 3'-O-isovaleryl-4'-O-angeloylkhellactone (paeruptorin C), or 3'-O-isovaleryl-4'-O-senecioylkhellactone] (peak 25)
Figure S27. ESI-QTof-MS spectrum of 3'-O-angeloyl-4'-O-(2-methyl butyryl)khellactone [3'-O-angeloyl-4'-O-isovaleryl khellactone, 3'-O-senecioyl-4'-O-(2-methyl butyryl)khellactone, or 3'-O-senecioyl-4'-O-isovaleryl khellactone] (peak 26)
Figure S28. ESI-QTof-MS spectrum of 3'-O-(2-methyl butyryl)-4'-O-(2-methyl butyryl)khellactone (or 3'-O-(2-methyl butyryl)-4'-O-isovalerylkhellactone) (peak 27)
Figure S29. ESI-QTof-MS spectrum of 3'-O-isovaleryl-4'-O-(2-methyl butyryl)khellactone (or 3'-O-isovaleryl-4'-O-isovalerylkhellactone) (peak 28)

Figure S30. Total scan PDA chromatograms of (a) flowers (S3), (b) roots (S15), (c) leaves (S9), and (d) stems (S21) of *Peucedanum japonicum*

Figure S31. UV-Vis spectra of all the peaks present in PDA chromatograms of the methanol extract of four parts of *Peucedanum japonicum* between 200–500 nm

Figure S32. Permutation plot for validation of OPLS-DA obtained from 200 permutation test.

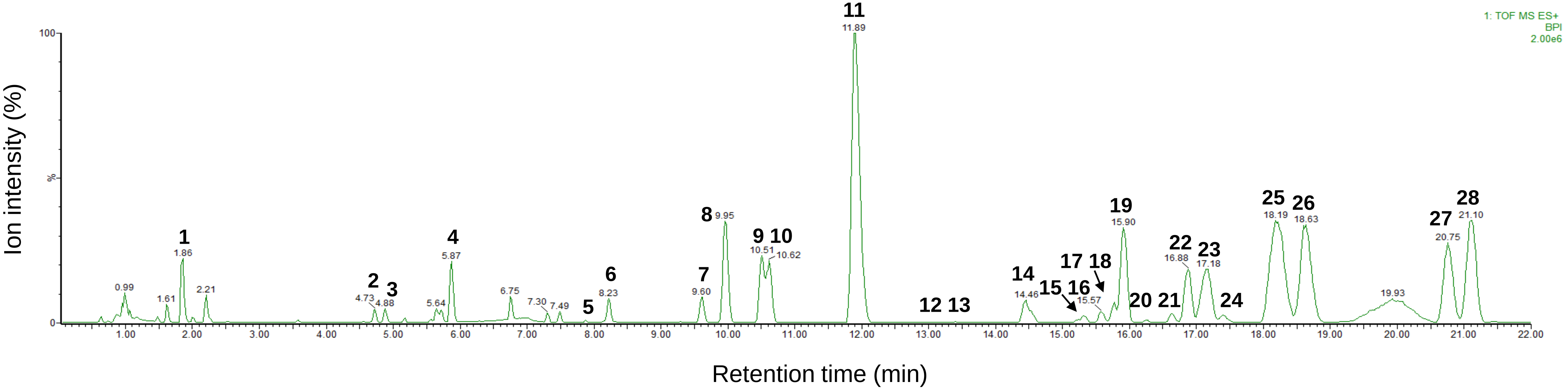

Figure S1. LC–MS base peak ion chromatograms of the dichloromethane fraction of the flowers of *P. japonicum* at positive ion mode (6 eV, ESI^+).

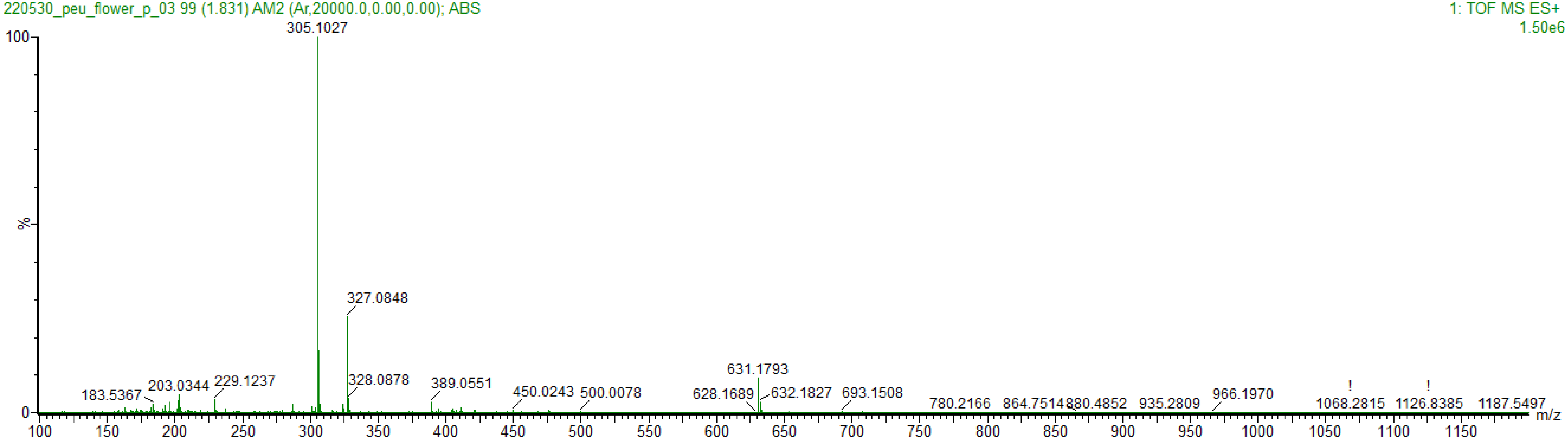

Figure S2. ESI-QTof-MS spectrum of oxypeucedanin hydrate (peak 1).

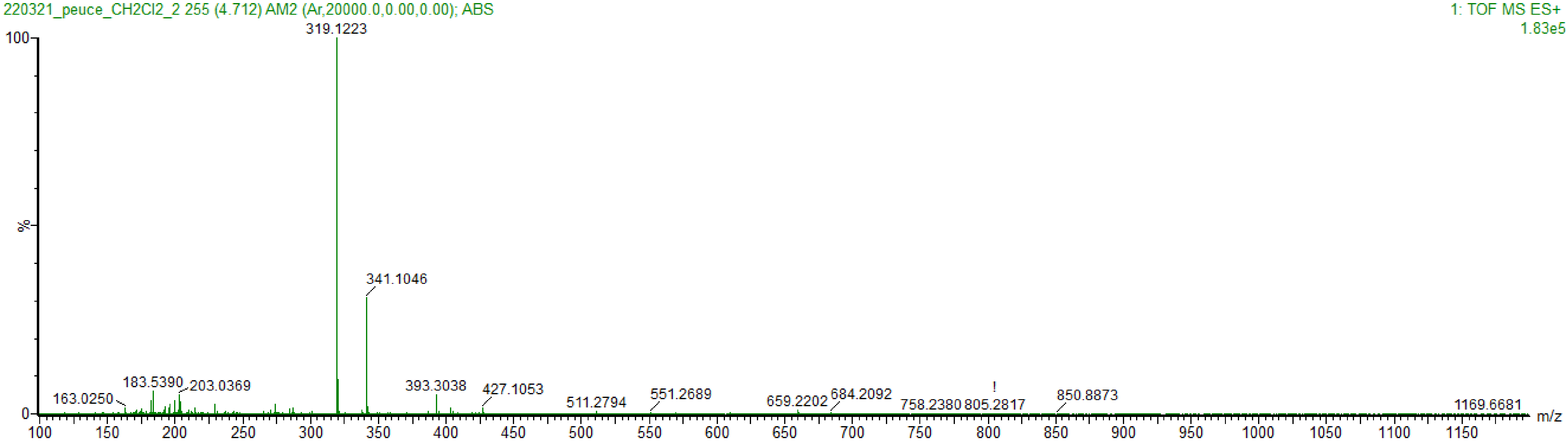

Figure S3. ESI-QTof-MS spectrum of oxypeucedanin methanolate (peak 2).

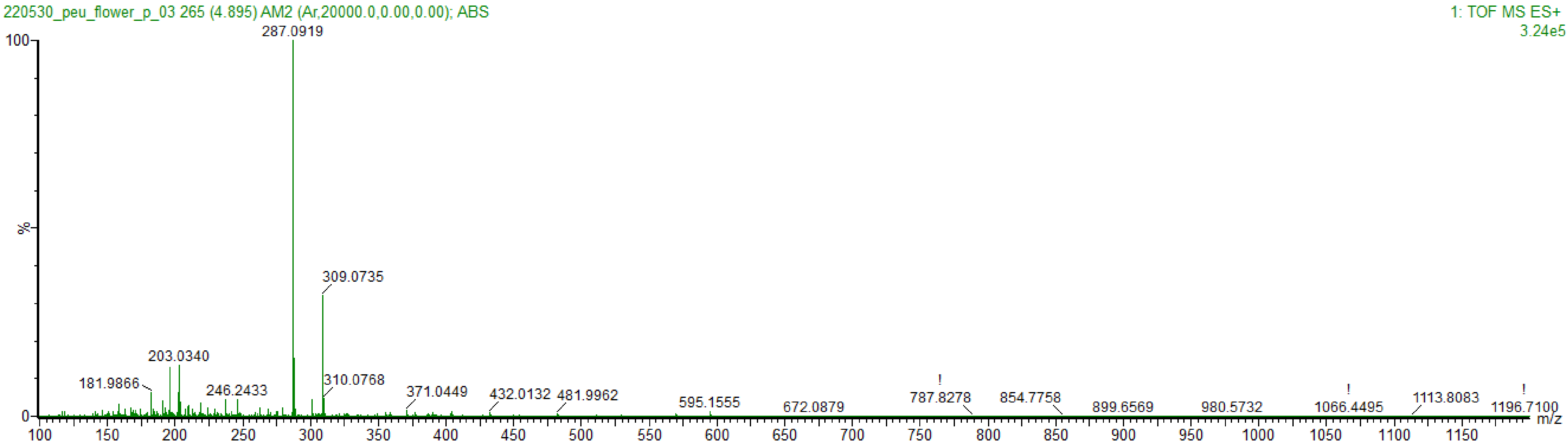

Figure S4. ESI-QTof-MS spectrum of pabulenol (peak 3).

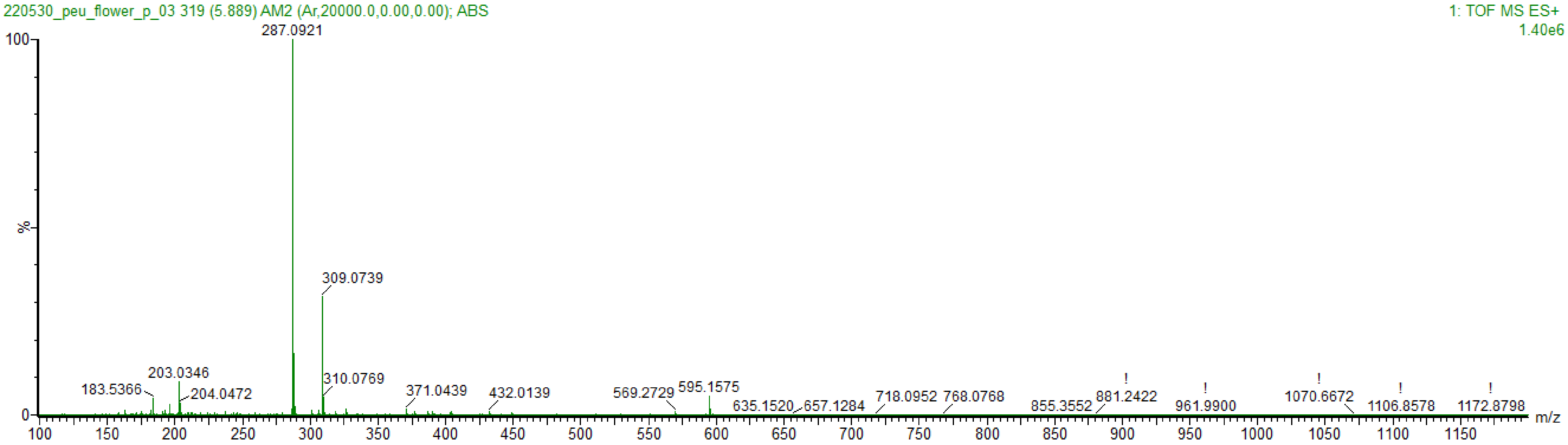

Figure S5. ESI-QTof-MS spectrum of 5-[(3-hydroxy-3-methyl-1-butenyl)oxy]psoralen (peak 4).

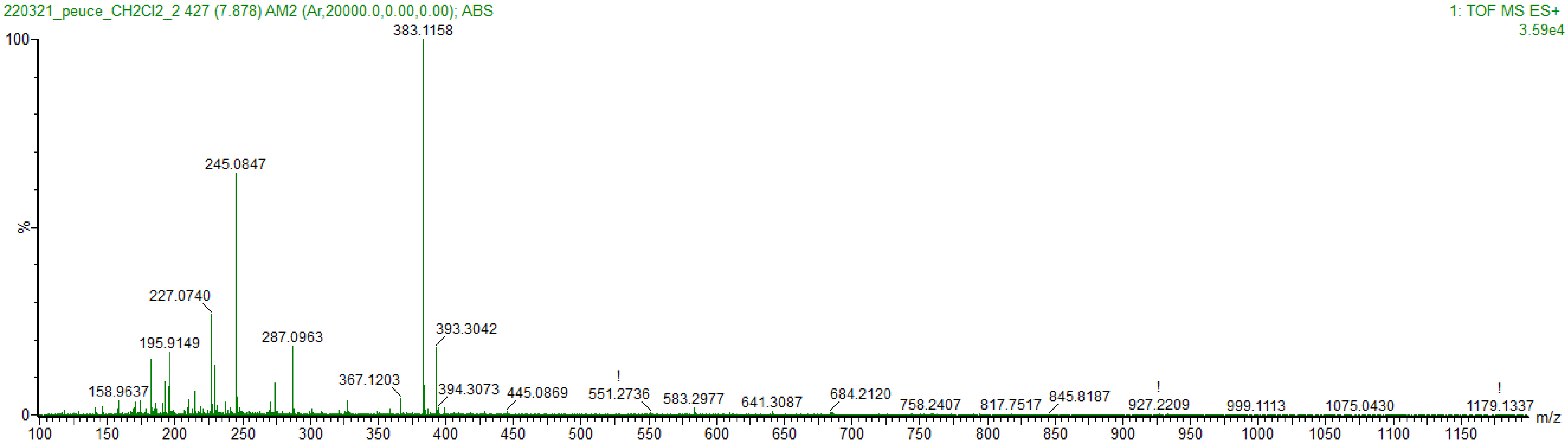

Figure S6. ESI-QTof-MS spectrum of 3'-O-acetyl-4'-O-propanoylkhellactone (peak 5).

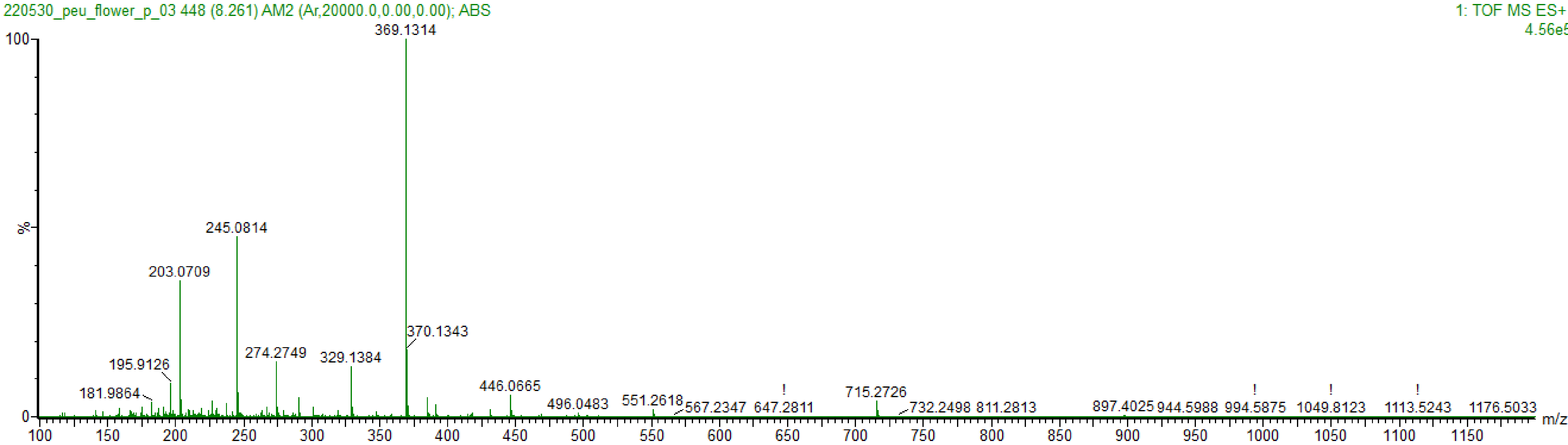

Figure S7. ESI-QTof-MS spectrum of 3'- O-(2-methyl-butyryl)-4'-hydroxy khellactone (or 3'-O-(isovaleryl)-4'-hydroxy khellactone) (peak 6)

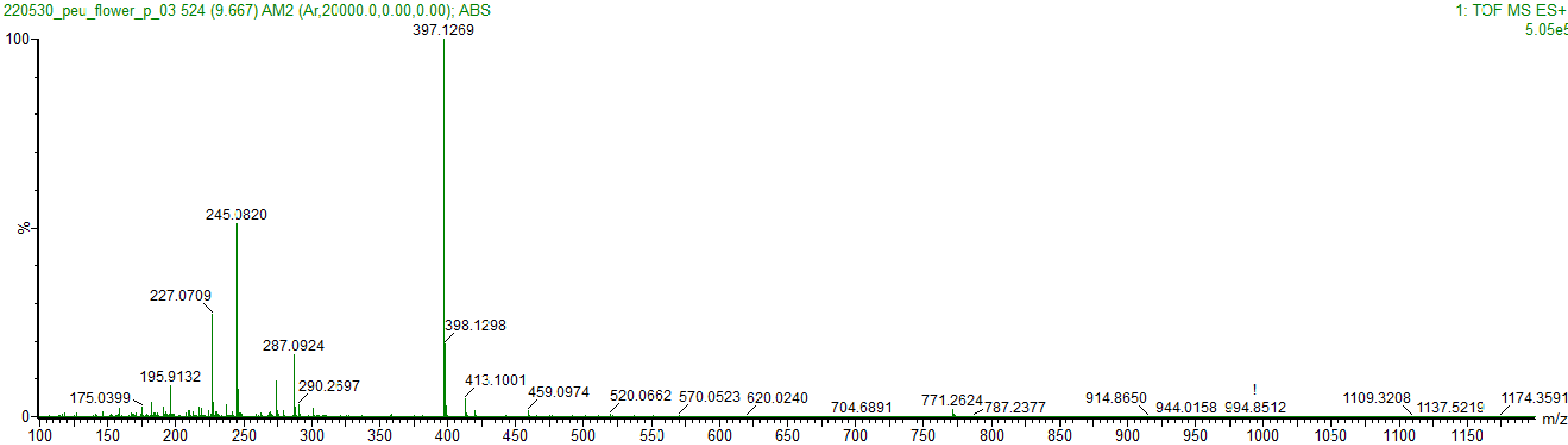

Figure S8. ESI-QTof-MS spectrum of 3'-O-acetyl-4'-O-isobutyryl khellactone (hyuganin D) (peak 7).

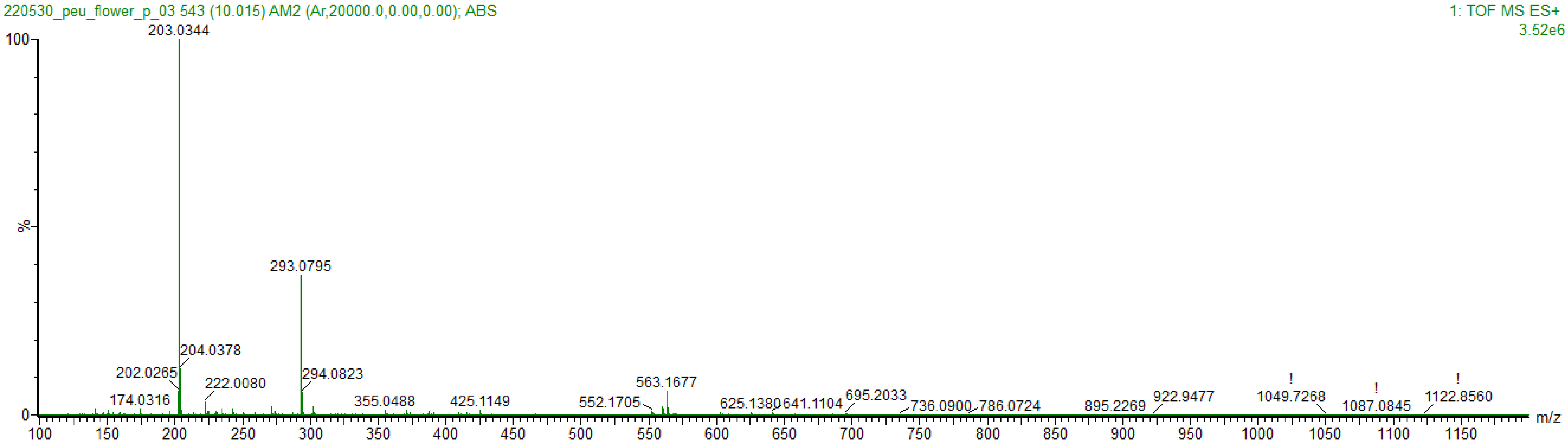

Figure S9. ESI-QTof-MS spectrum of isoimpertorin (peak 8).

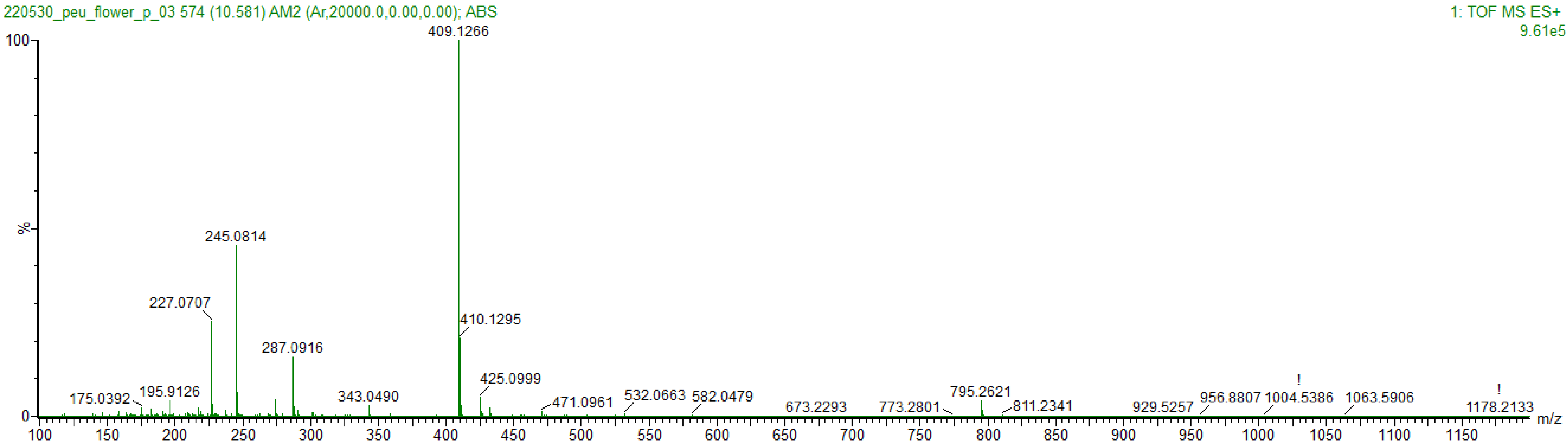

Figure S10. ESI-QTof-MS spectrum of 3'-O-acetyl-4'-O-angeloylkhellactone (pteryxin) (peak 9).

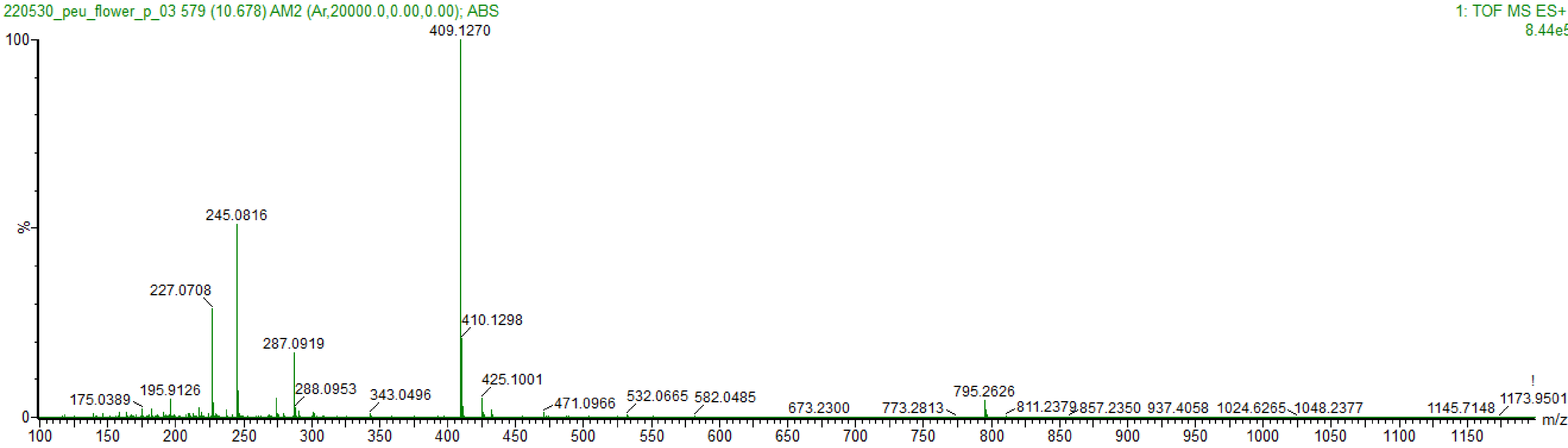

Figure S11. ESI-QTof-MS spectrum of 3'-O-acetyl-4'-O-senecioylkhellactone (peak 10).

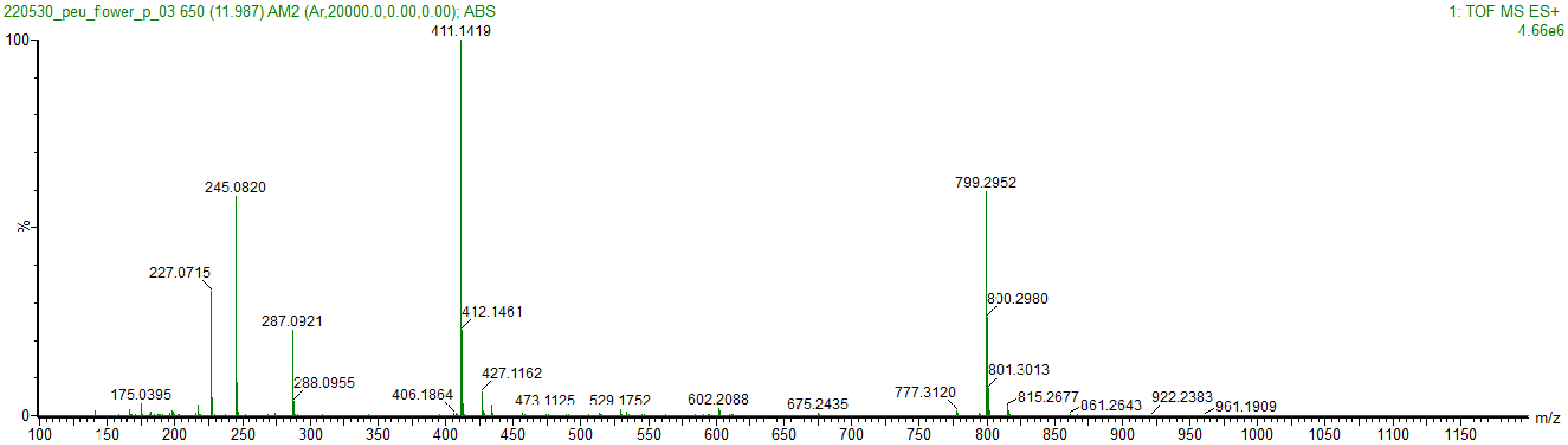

Figure S12. ESI-QTof-MS spectrum of 3'-O-acetyl-4'-O-(2-methyl butanoate)khellactone (or 3'-O-acetyl-4'-O-isovalerylkhellactone) (peak 11).

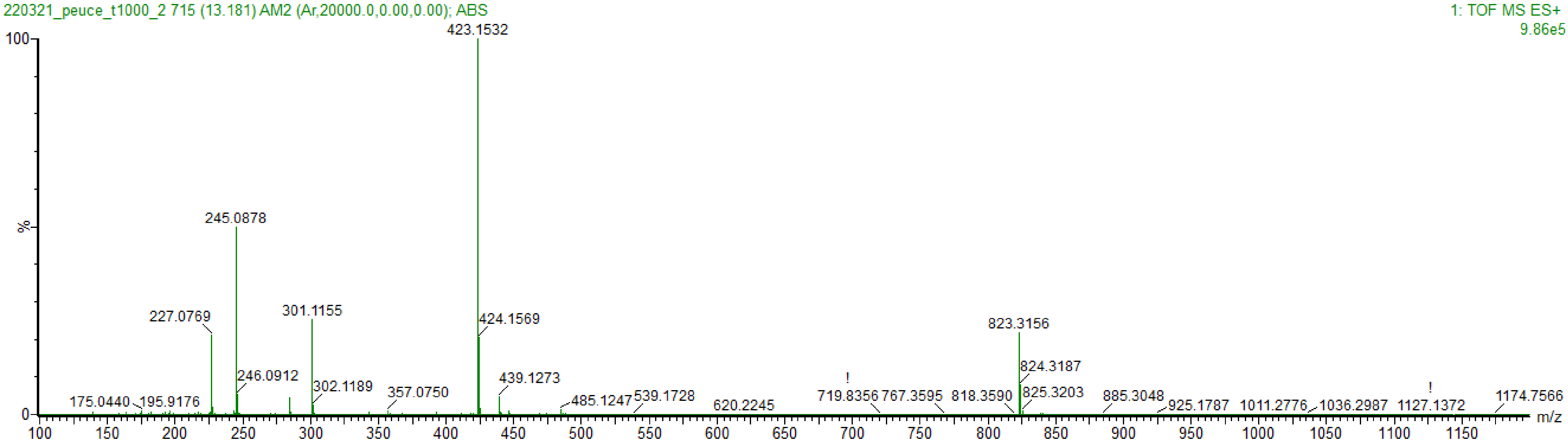

Figure S13. ESI-QTof-MS spectrum of 3'-O-propanoyl-4'-O-angeloyl khellactone (or 3'-O-propanoyl-4'-O-senecioylkhellactone) (peak 12).

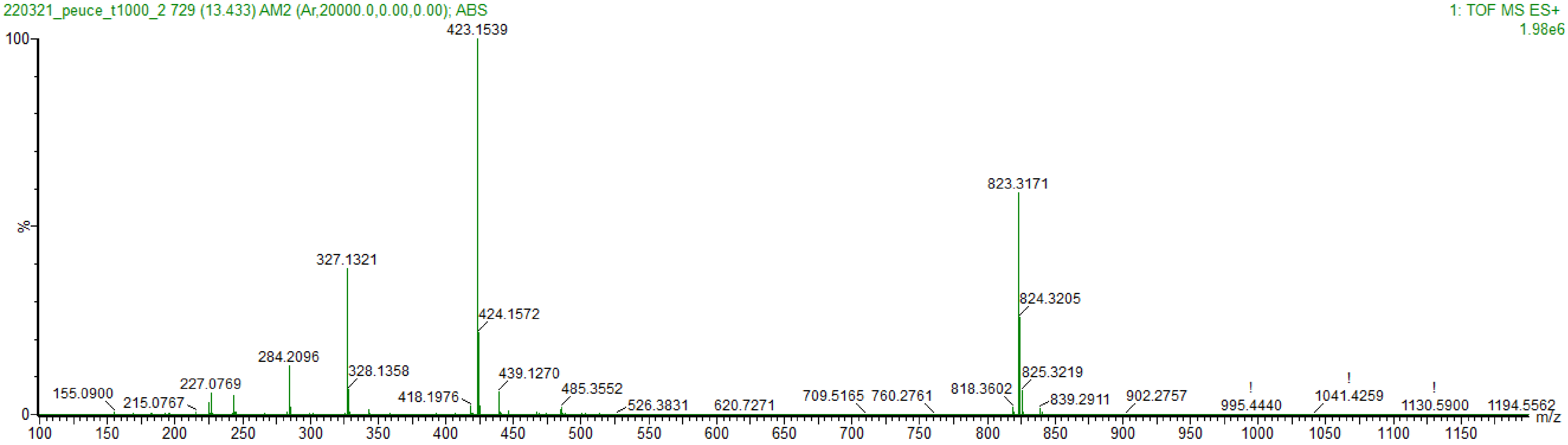

Figure S14. ESI-QTof-MS spectrum of 3'-O-angeloyl-4'-O-propanoylkhellactone (or 3'-O-senecioyl-4'-O-propanoylkhellactone) (peak 13).

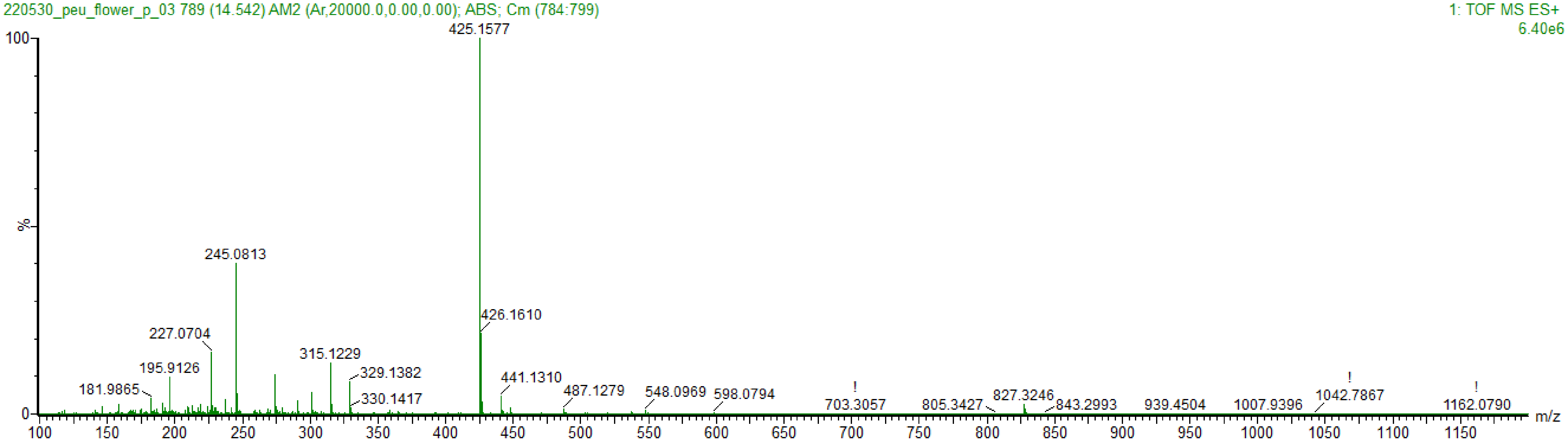

Figure S15. ESI-QTof-MS spectrum of 3'-O-isobutyryl-4'-O-isobutyrylkhellactone (peak 14).

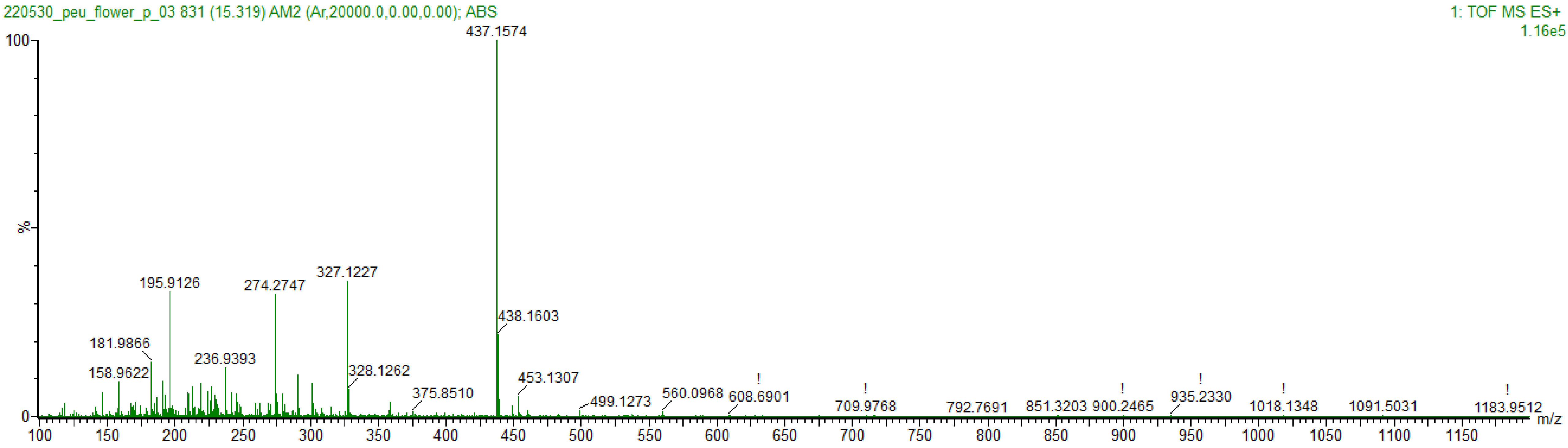

Figure S16. ESI-QTof-MS spectrum of 3'-O-angeloyl-4'-O-isobutyrylkhellactone (peak 15).

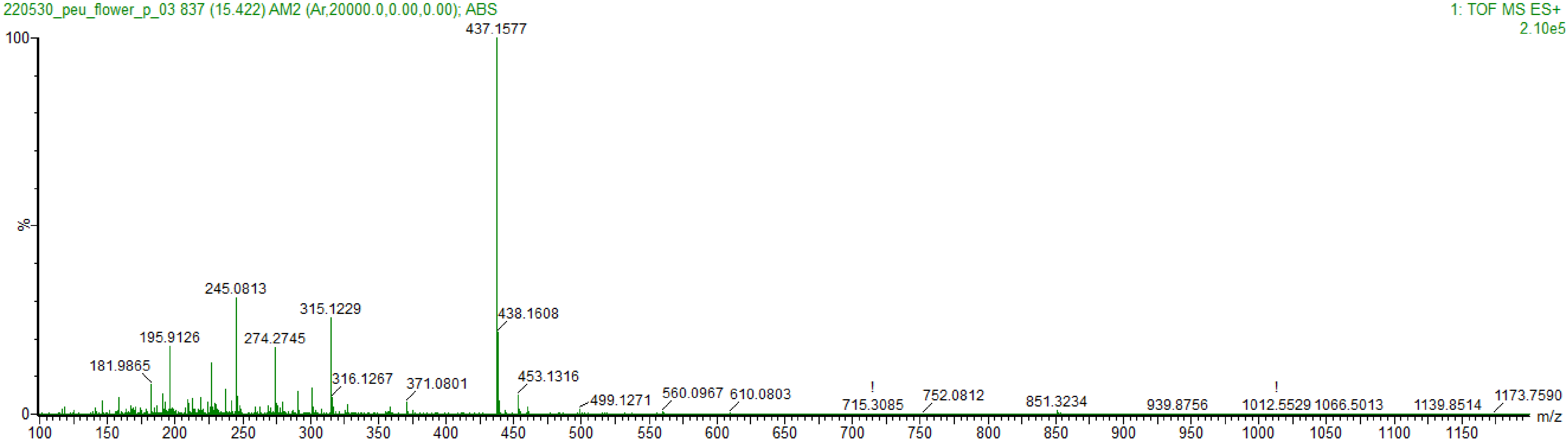

Figure S17. ESI-QTof-MS spectrum of 3'-O- isobutyryl-4'-O-angeloylkhellactone (peak 16).

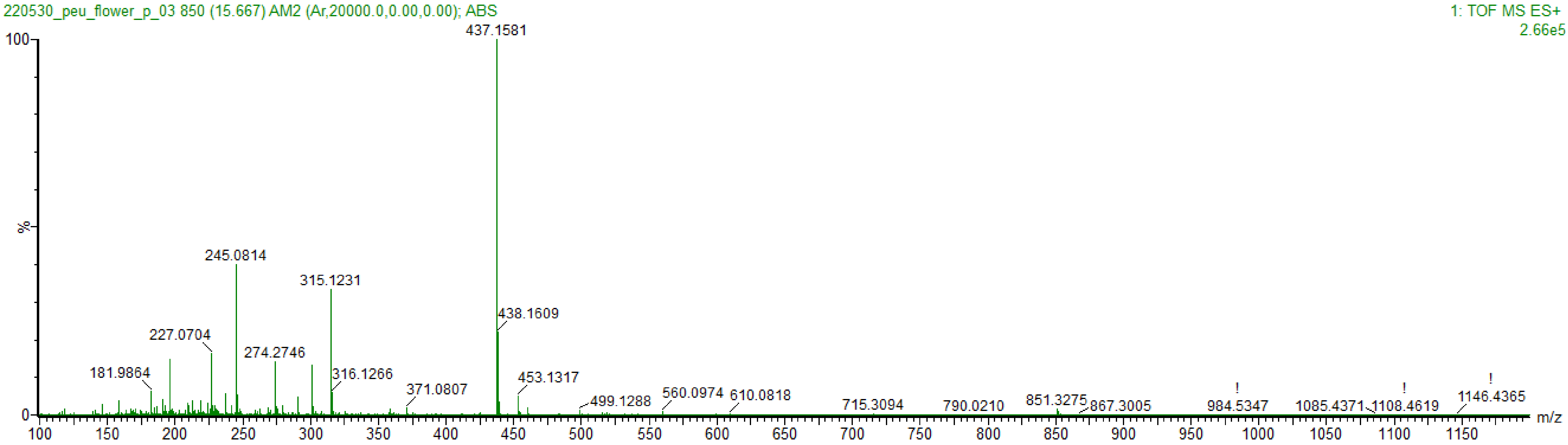

Figure S18. ESI-QTof-MS spectrum of 3'-O-isobutyryl-4'-O-senecioylkhellactone (peak 17).

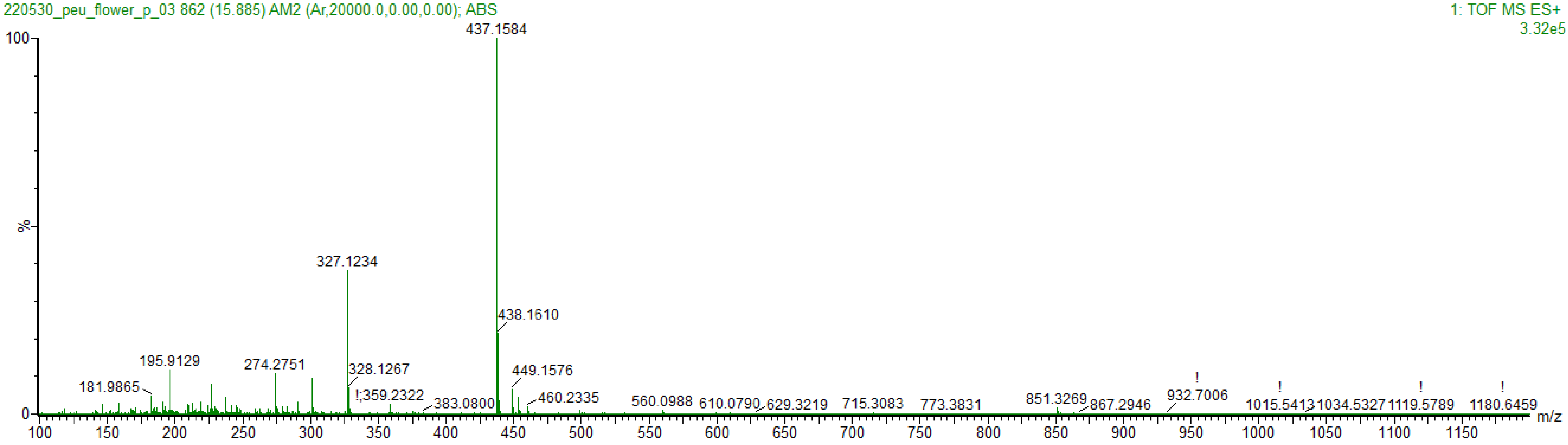

Figure S19. ESI-QTof-MS spectrum of 3'-O-senecioyl-4'-O-isobutyrylkhellactone (peak 18).

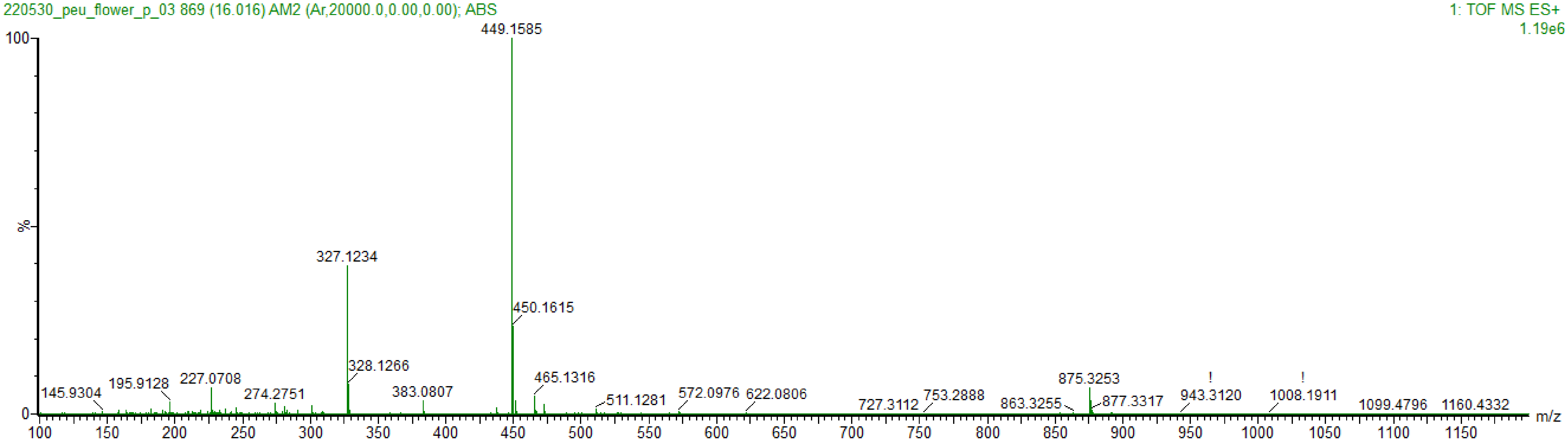

Figure S20. ESI-QTof-MS spectrum of 3'-O-angeloyl-4'-O-angeloylkhellactone (paeruptorin B) (peak 19).

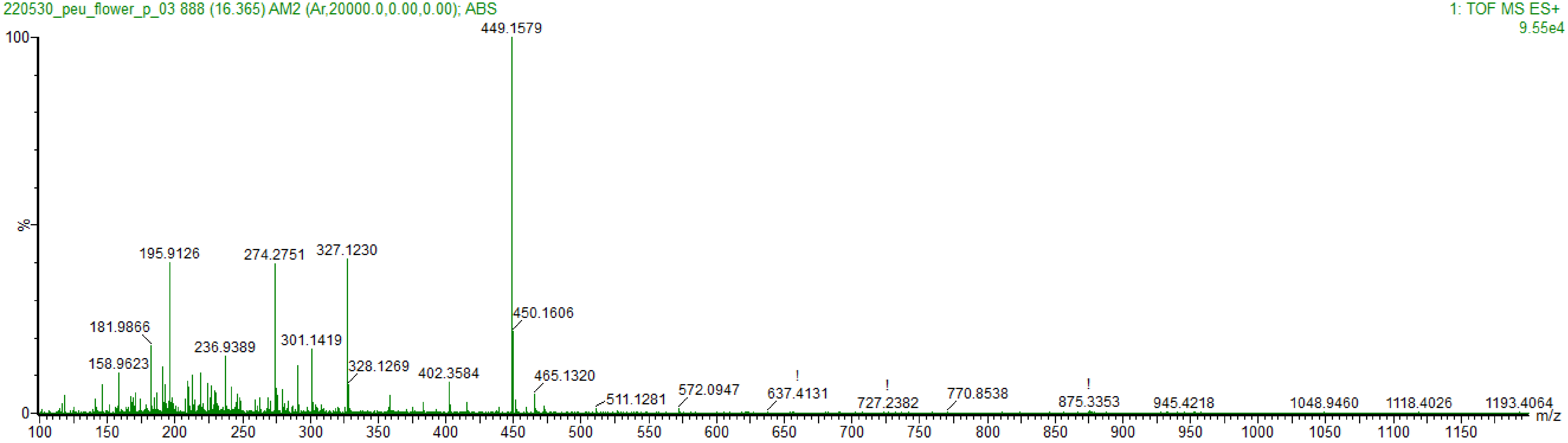

Figure S21. ESI-QTof-MS spectrum of 3'-O-angeloyl-4'-O-senecioylkhellactone (peak 20).

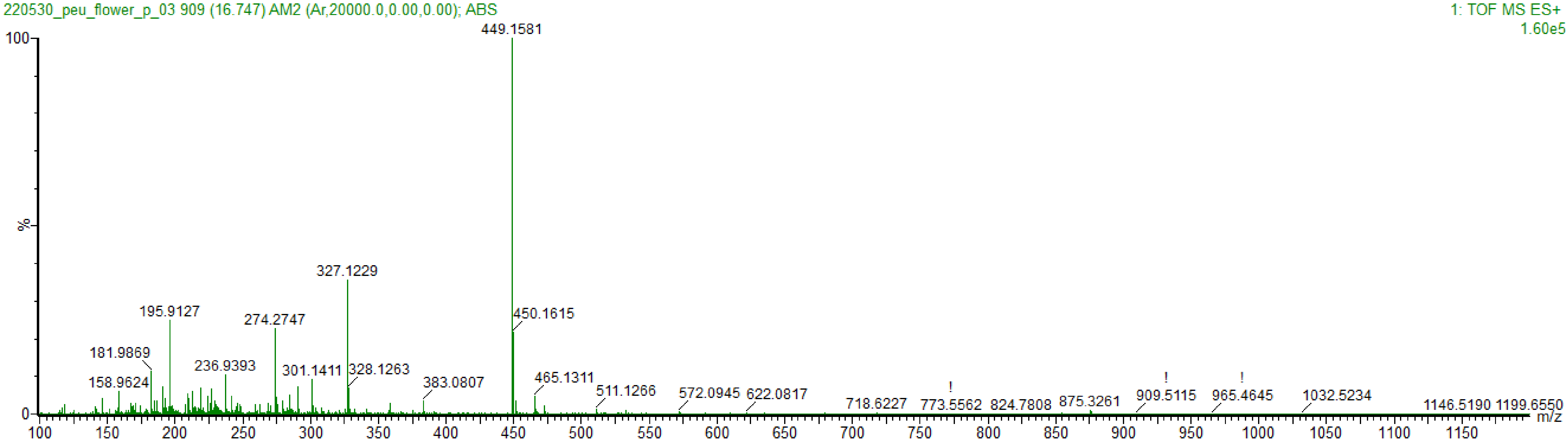

Figure S22. ESI-QTof-MS spectrum of 3'-O-senecioyl-4'-O-senecioylkhellactone (peak 21).

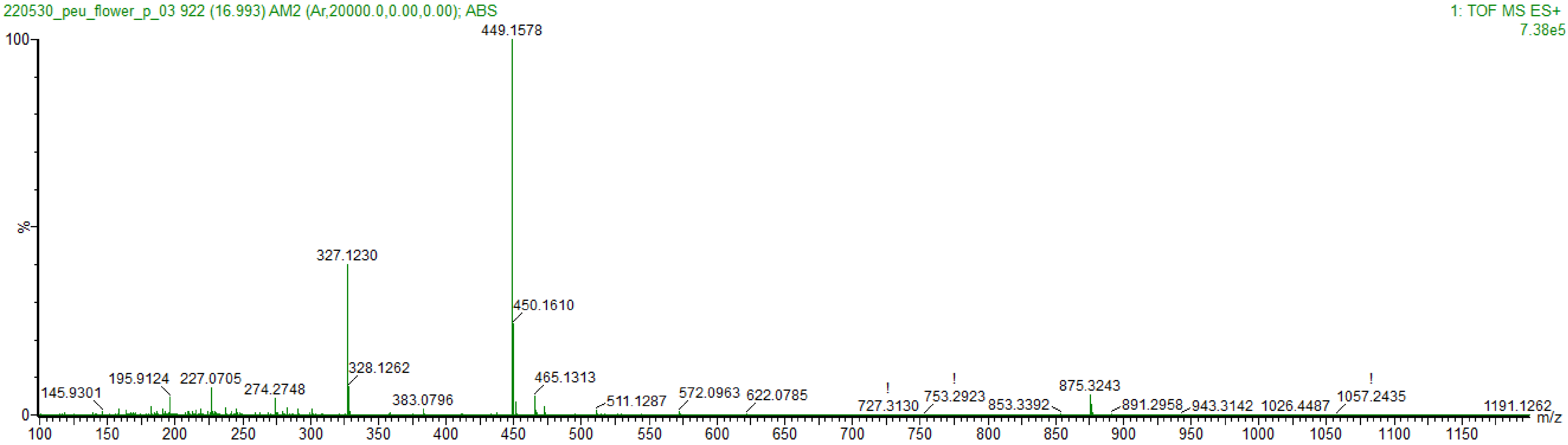

Figure S23. ESI-QTof-MS spectrum of 3'-O-senecioyl-4'-O-angeloylkhellactone (peak 22).

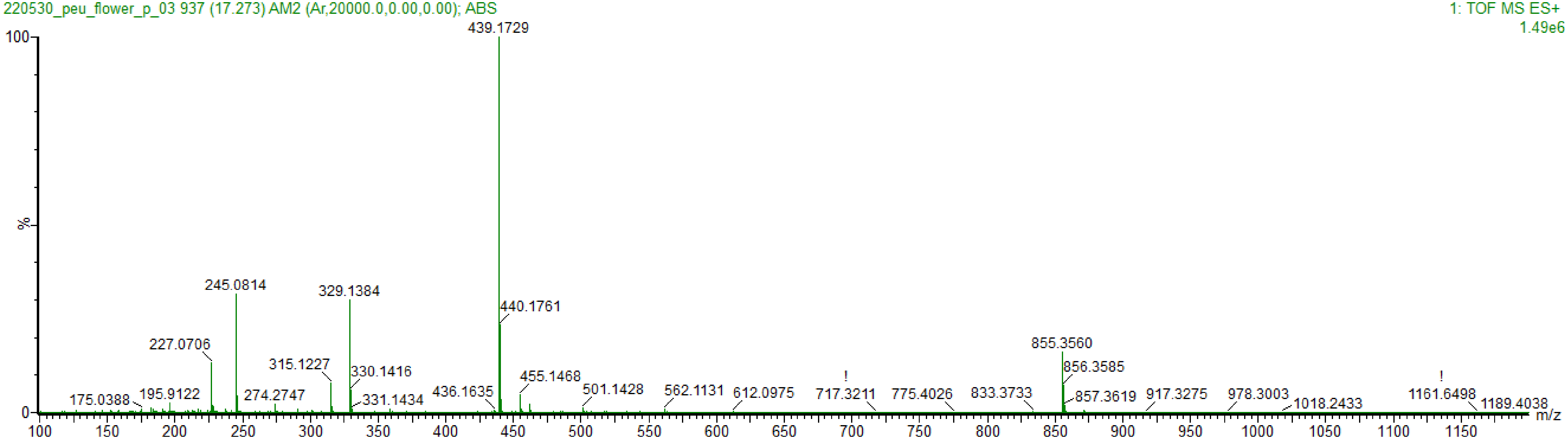

Figure S24. ESI-QTof-MS spectrum of 3'-O-(2-methyl butyryl)-4'-O-isobutyrylkhellactone (peak23).

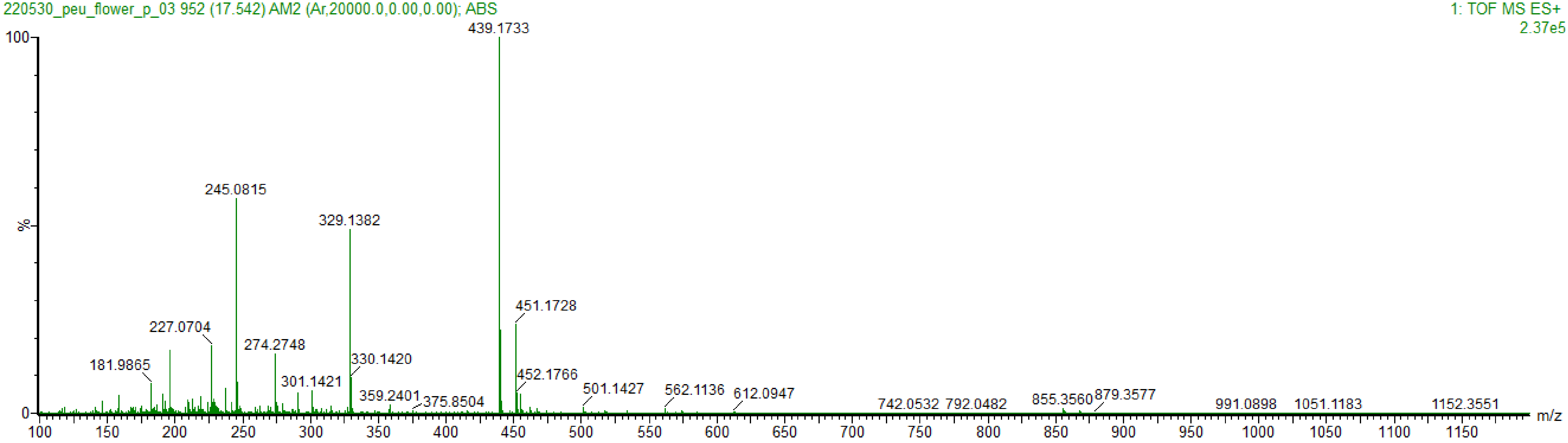

Figure S25. ESI-QTof-MS spectrum of 3'-O-isovaleryl-4'-O-isobutyrylkhellactone (peak 24).

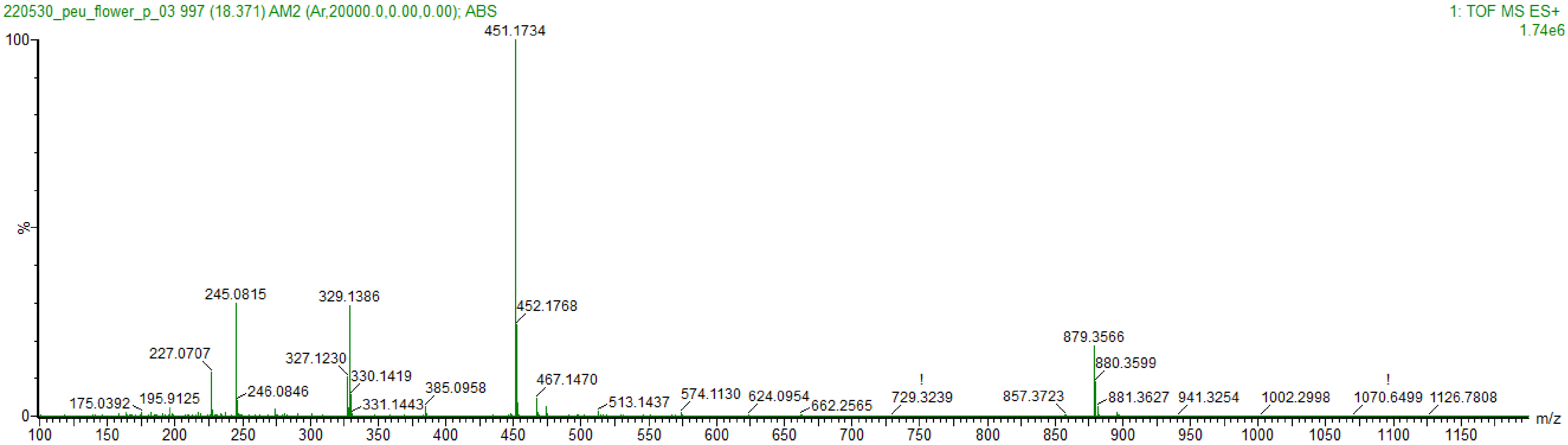

Figure S26. ESI-QTof-MS spectrum of 3'-O-(2-methyl butyryl)-4'-O-angeloylkhellactone [3'-O-(2-methyl butyryl)-4'-O-senecioyl khellactone, 3'-O-isovaleryl-4'-O-angeloylkhellactone (paeruptorin C), or 3'-O-isovaleryl-4'-O-senecioylkhellactone] (peak 25).

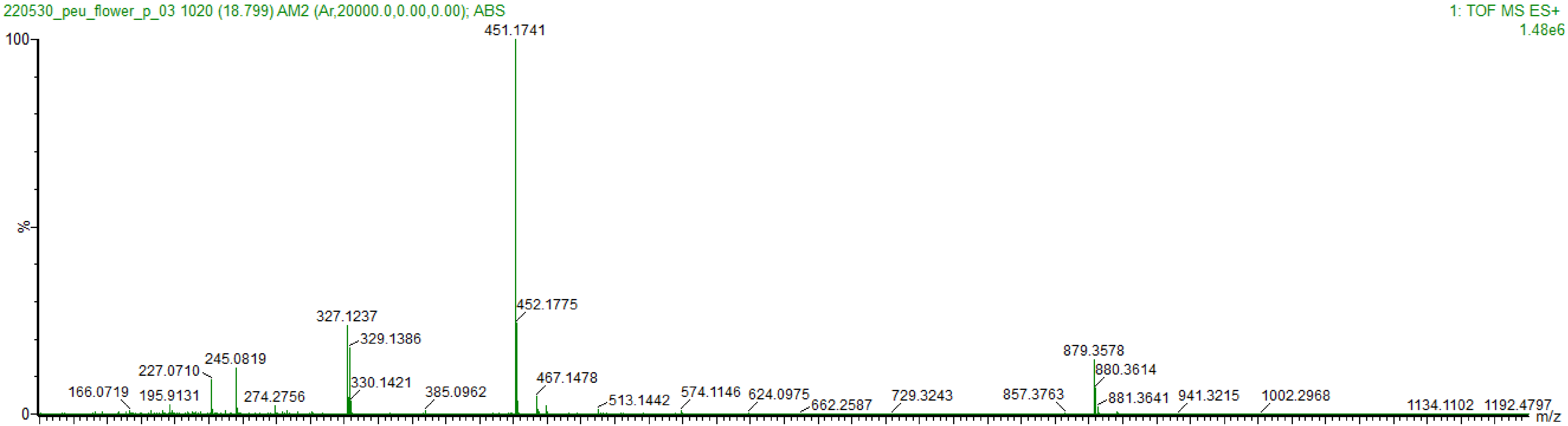

Figure S26. ESI-QTof-MS spectrum of 3'-O-angeloyl-4'-O-(2-methyl butyryl)khellactone [3'-O-angeloyl-4'-O-isovaleryl khellactone, 3'-O-senecioyl-4'-O-(2-methyl butyryl)khellactone, or 3'-O-senecioyl-4'-O-isovaleryl khellactone] (peak 26).

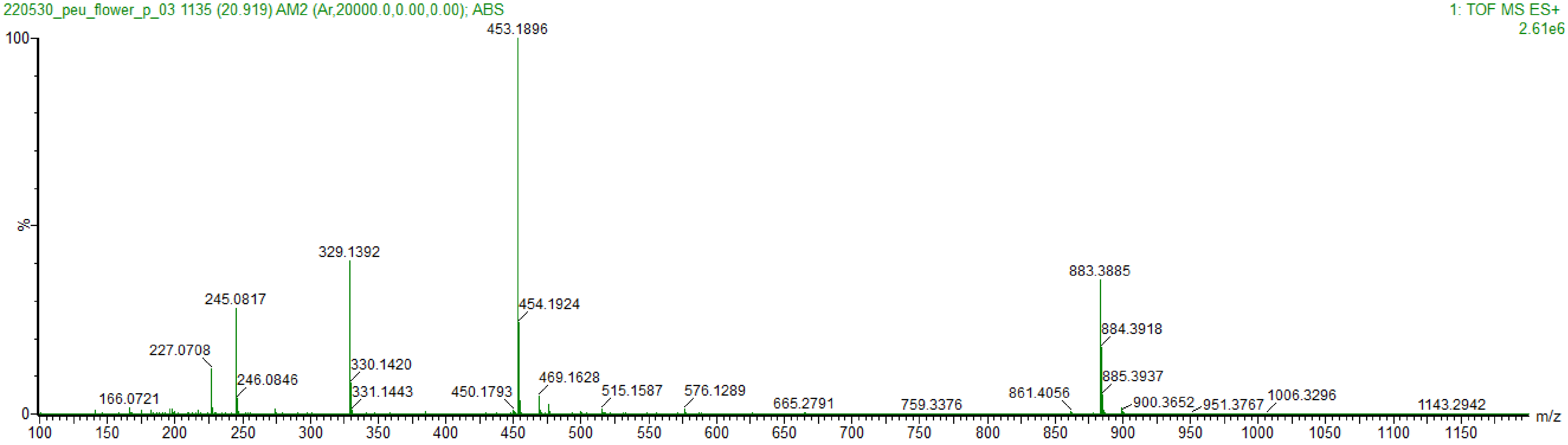

Figure S28. ESI-QTof-MS spectrum of 3'-O-(2-methyl butyryl)-4'-O-(2-methyl butyryl)khellactone (or 3'-O-(2-methyl butyryl)-4'-O-isovalerylkhellactone) (peak 27).

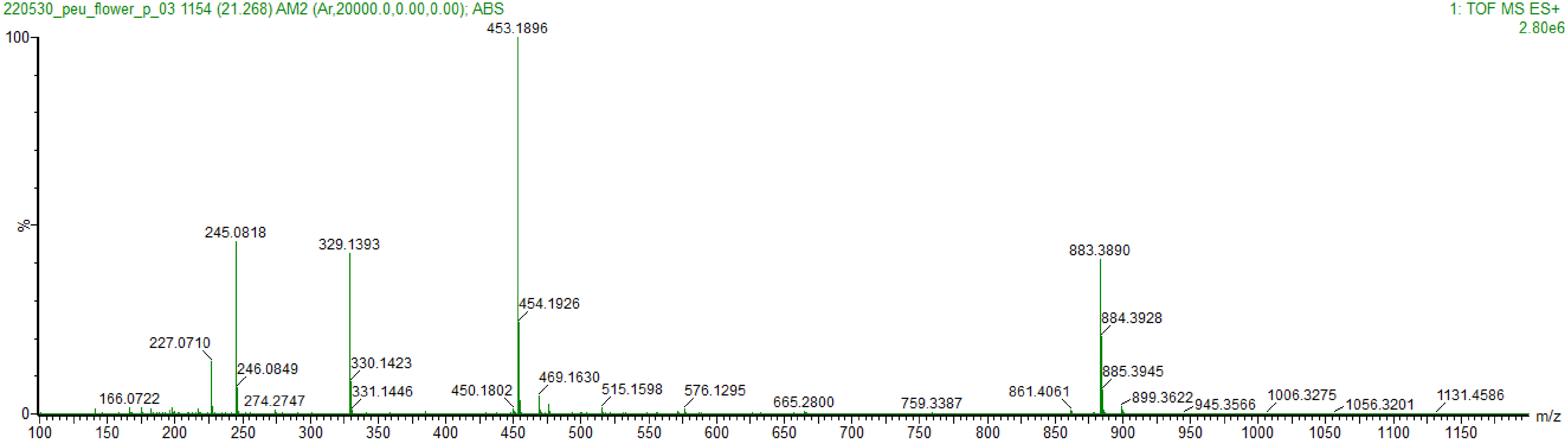

Figure S29. ESI-QTof-MS spectrum of 3'-O-isovaleryl-4'-O-(2-methyl butyryl)khellactone (or 3'-O-isovaleryl-4'-O-isovalerylkhellactone) (peak 28).

(a)
(b)
(c)
(d)

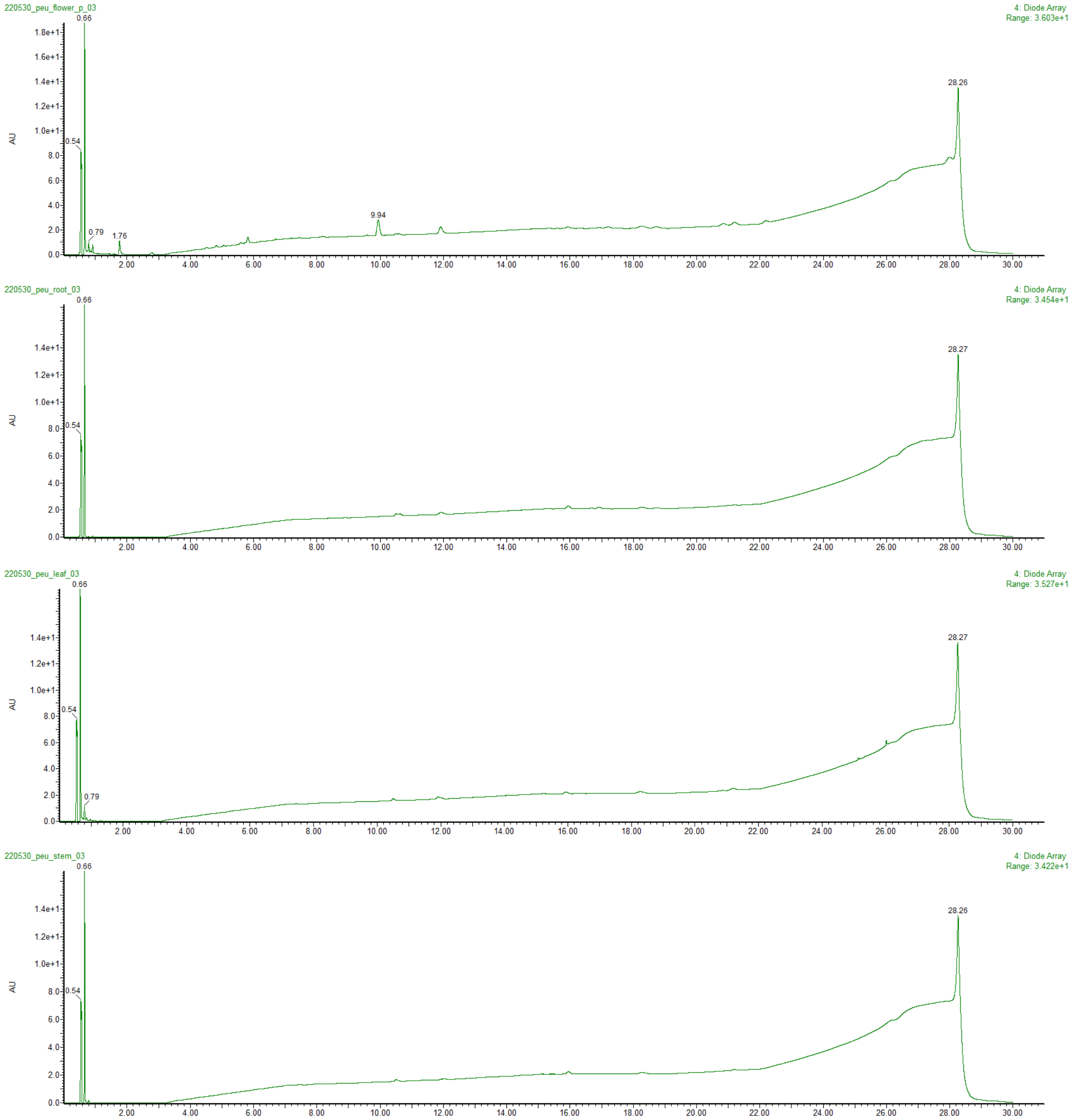

Figure S30. Total scan PDA chromatograms of (**a**) flowers (S3), (**b**) roots (S15), (**c**) leaves (S9), and (**d**) stems (S21) of *Peucedanum japonicum*

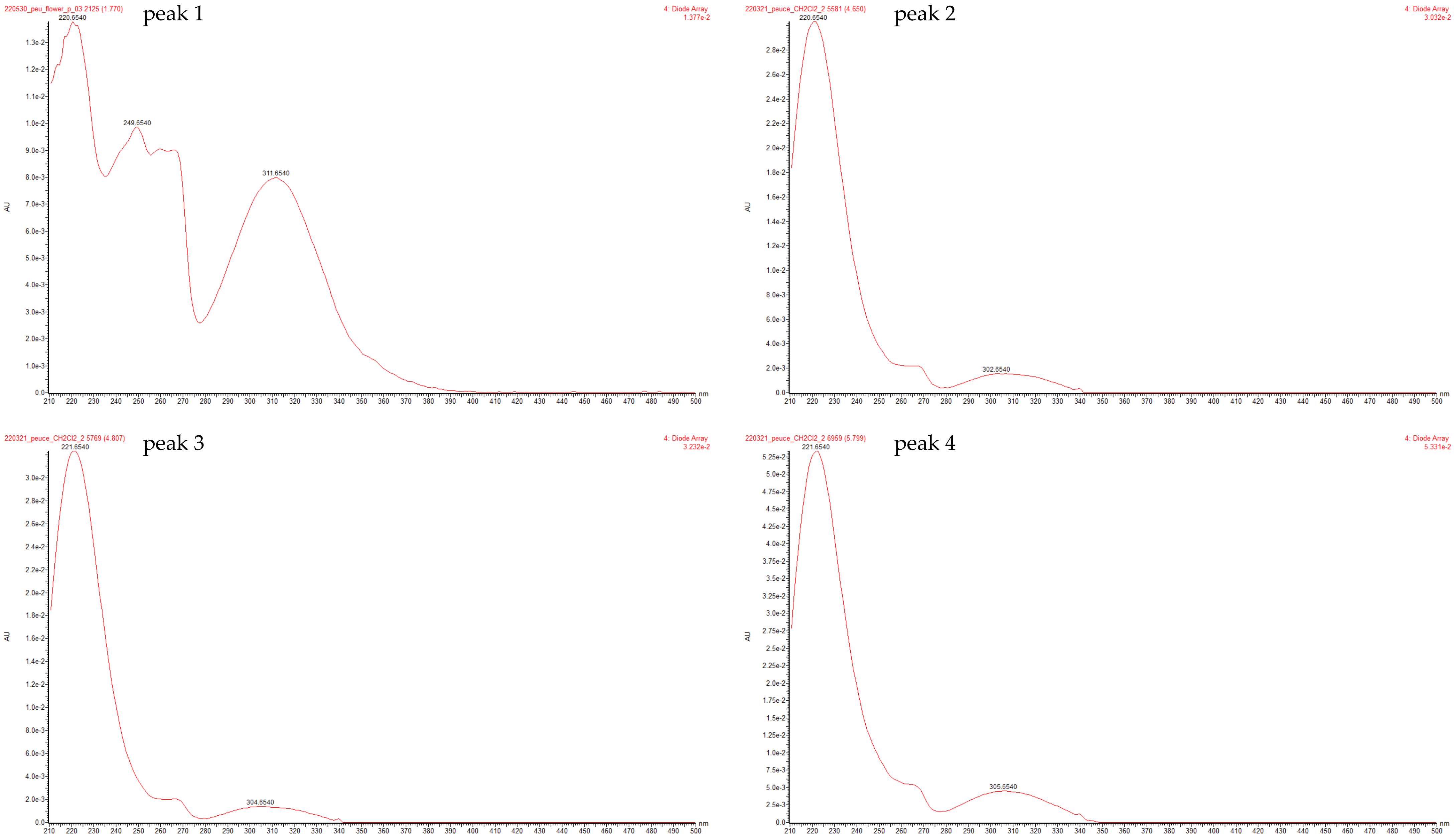

Figure S31. UV-Vis spectra of all the peaks present in PDA chromatograms of the methanol extract of four parts of *Peucedanum japonicum* between 200–500 nm.

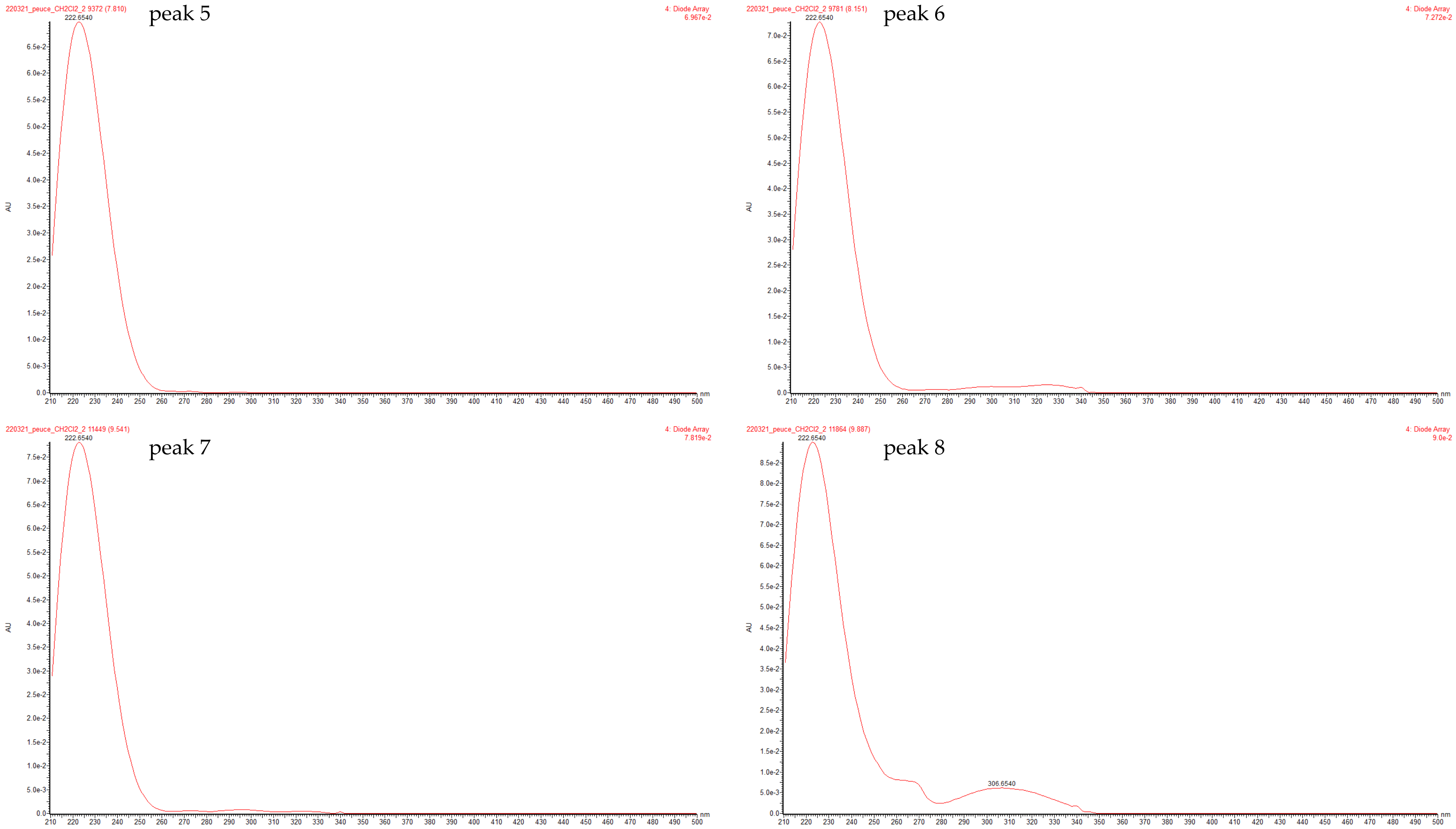

Figure S31. UV-Vis spectra of all the peaks present in PDA chromatograms of the methanol extract of four parts of *Peucedanum japonicum* between 200–500 nm. (*Cont*)

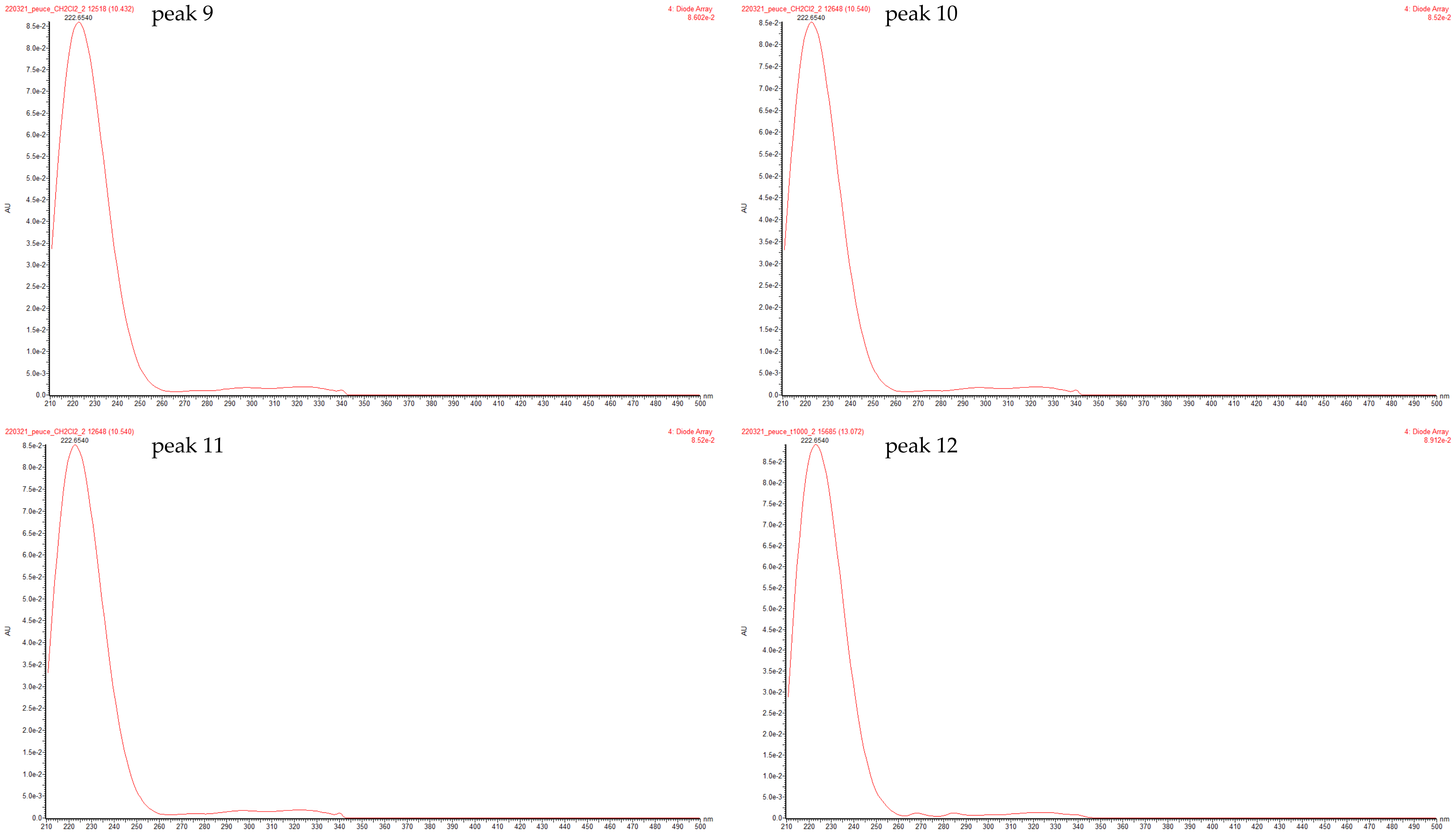

Figure S31. UV-Vis spectra of all the peaks present in PDA chromatograms of the methanol extract of four parts of *Peucedanum japonicum* between 200–500 nm. (*Cont*)

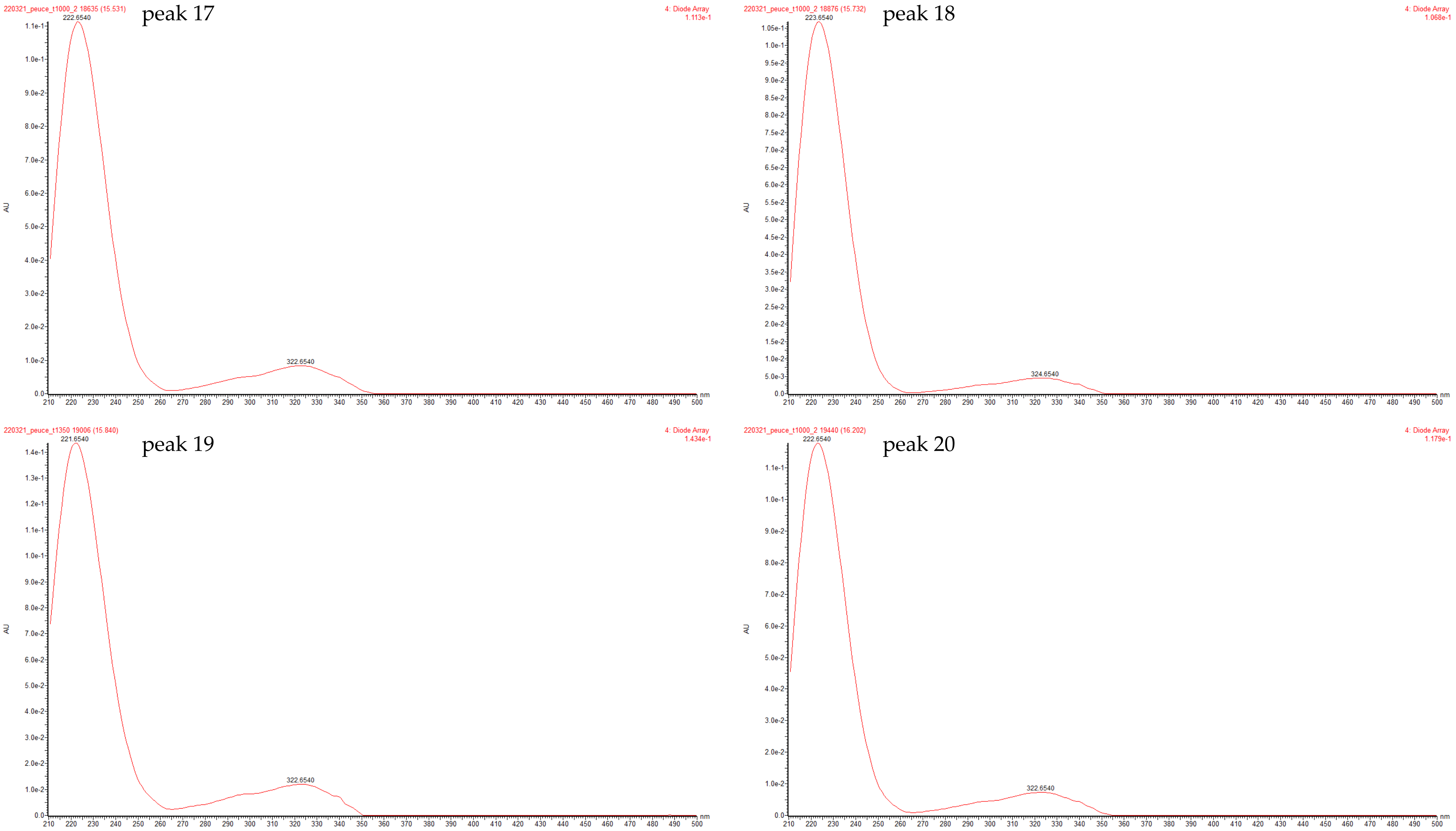

Figure S31. UV-Vis spectra of all the peaks present in PDA chromatograms of the methanol extract of four parts of *Peucedanum japonicum* between 200–500 nm. (*Cont*)

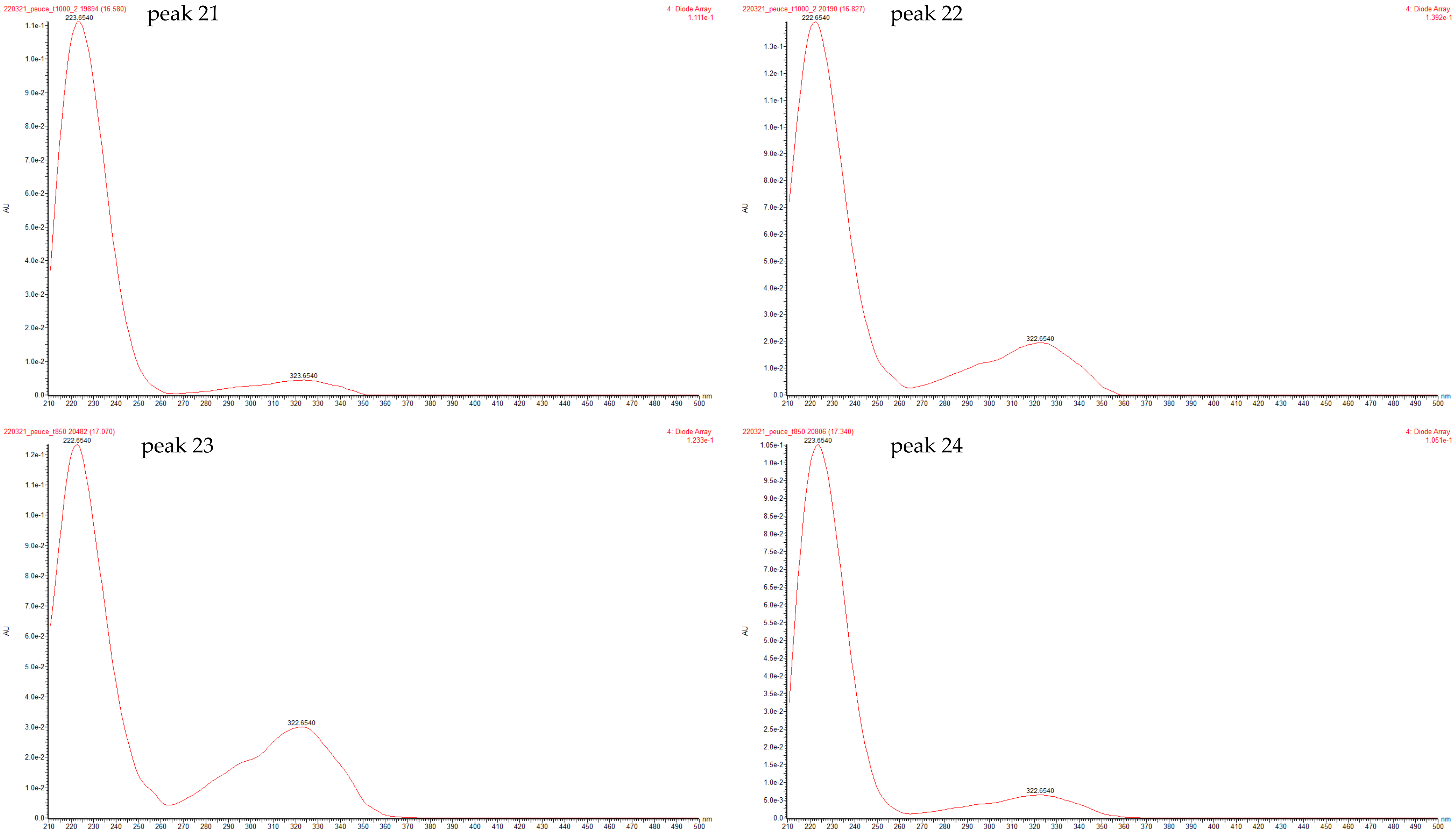

Figure S31. UV-Vis spectra of all the peaks present in PDA chromatograms of the methanol extract of four parts of *Peucedanum japonicum* between 200–500 nm. (*Cont*)

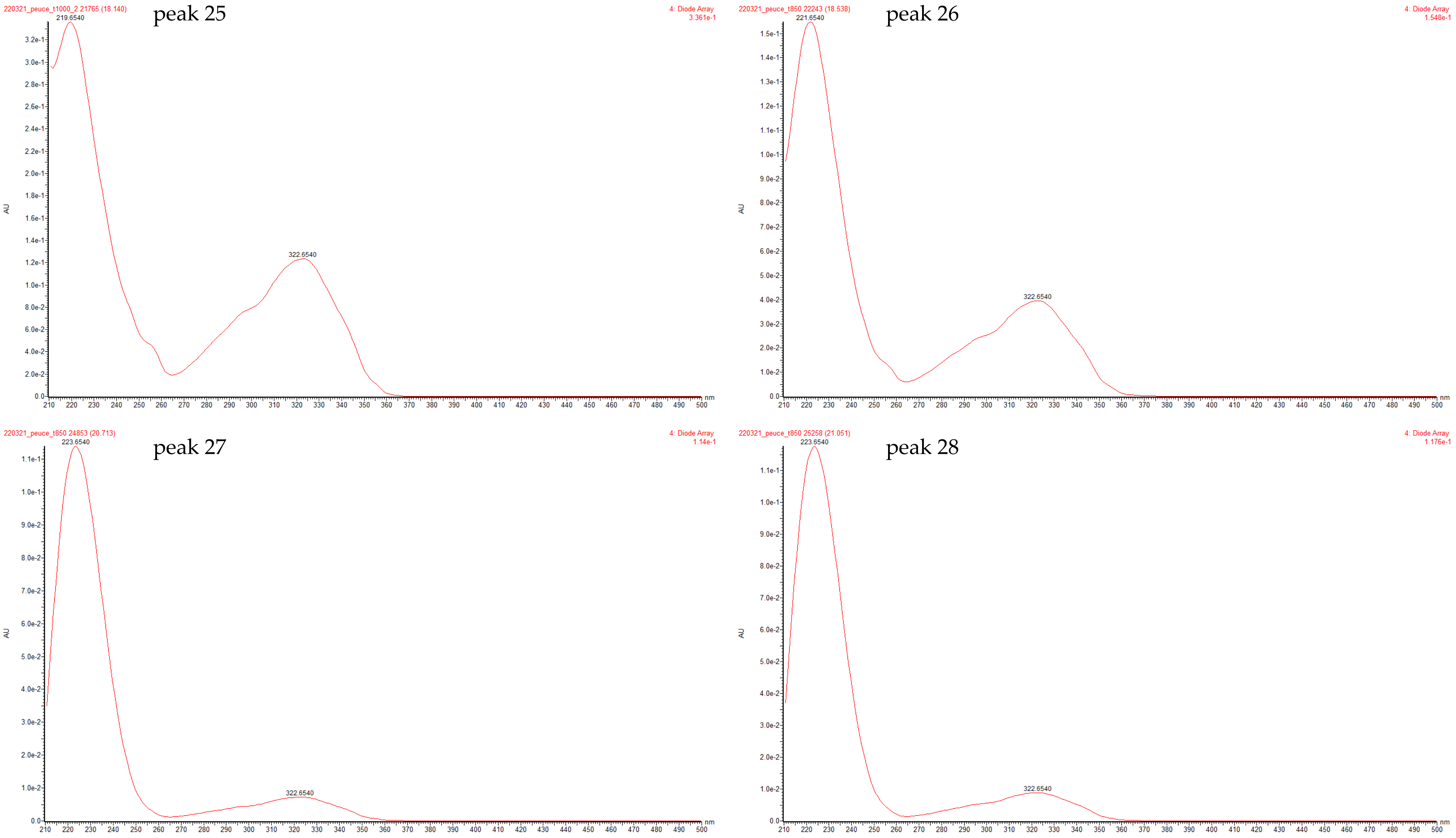

Figure S31. UV-Vis spectra of all the peaks present in PDA chromatograms of the methanol extract of four parts of *Peucedanum japonicum* between 200–500 nm. (*Cont*)

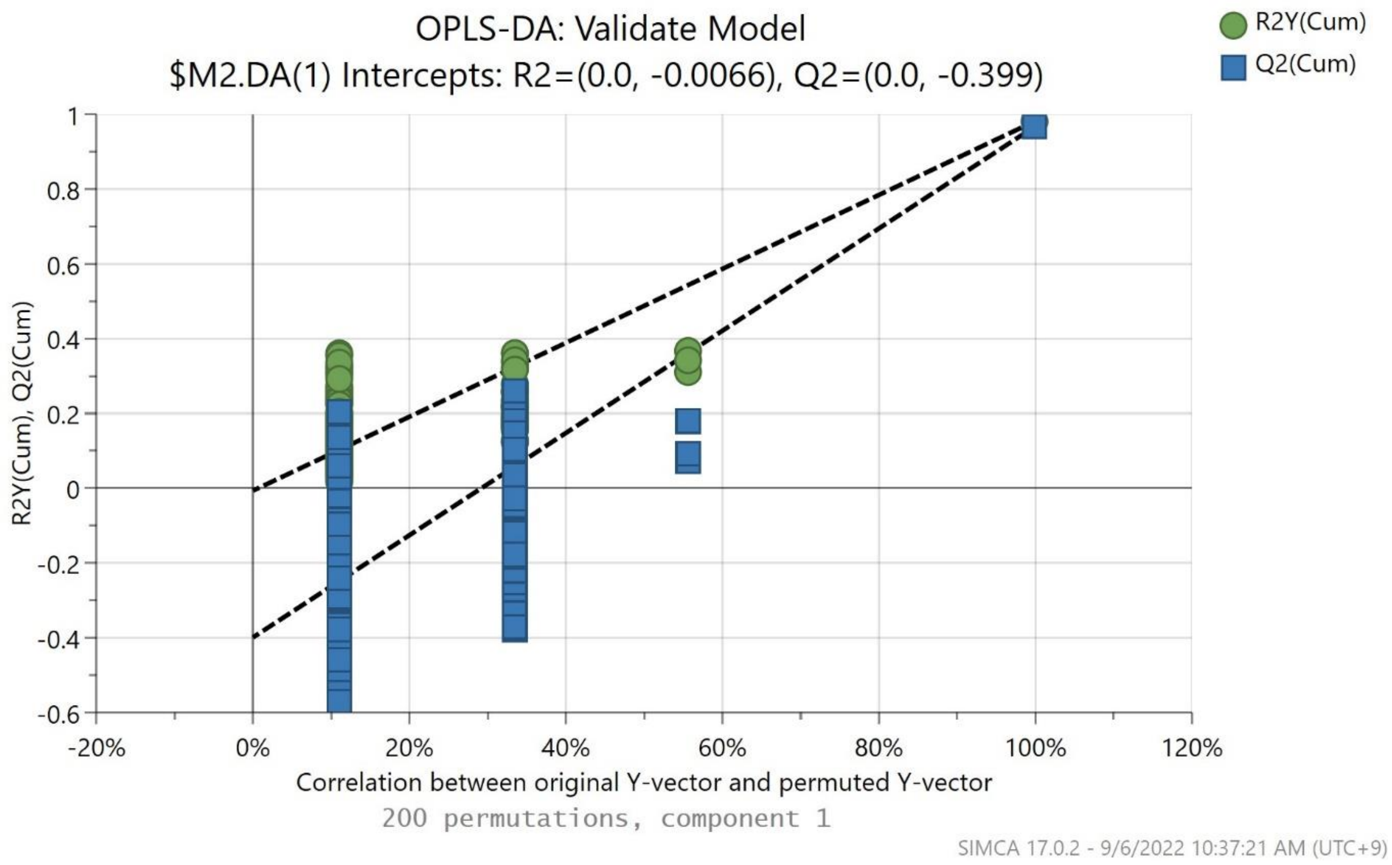

Figure S32. Permutation plot for validation of OPLS-DA obtained from 200 permutation test.
